# Methamphetamine (MA) use and MA-induced psychosis are associated with increasing aberrations in the compensatory immunoregulatory system, interleukin-1α, and CCL5 levels

**DOI:** 10.1038/s41398-023-02645-6

**Published:** 2023-11-23

**Authors:** Rasmon Kalayasiri, Kanokwan Dadwat, Supaksorn Thika, Sunee Sirivichayakul, Michael Maes

**Affiliations:** 1 Department of Psychiatry, Epidemiology of Psychiatric Disorders and Mental Health Research Unit, Faculty of Medicine, Chulalongkorn University, Bangkok, Thailand; 2 King Chulalongkorn Memorial Hospital, Thai Red Cross Society, Bangkok, Thailand; 3 Division of Allergy and Clinical Immunology, Department of Medicine, Faculty of Medicine, Chulalongkorn University, Bangkok, Thailand; 4 Cognitive Fitness and Technology Research Unit, Faculty of Medicine, Chulalongkorn University, Bangkok, Thailand; 5 Department of Psychiatry, Medical University of Plovdiv, Plovdiv, Bulgaria; 6 Research Institute, Medical University Plovdiv, Plovdiv, Bulgaria; 7 Kyung Hee University, 26 Kyungheedae-ro, Dongdaemun-gu, Seoul, 02447 Korea; 8 Sichuan Provincial Center for Mental Health, Sichuan Provincial People’s Hospital, School of Medicine, University of Electronic Science and Technology of China, Chengdu, 610072 China; 9 Key Laboratory of Psychosomatic Medicine, Chinese Academy of Medical Sciences, Chengdu, 610072 China

**Keywords:** Molecular neuroscience, Addiction

## Abstract

There are only a few studies reporting on the immunological profiles of methamphetamine (MA) use, MA dependency, or MA-induced psychosis (MAP). This study measured M1 macrophage, T helper (Th)-1, Th-2, growth factor, and chemokine profiles, as well as the immune inflammatory response system (IRS) and compensatory immunoregulatory system (CIRS) in peripheral blood samples from patients with MA use (*n* = 51), MA dependence (*n* = 47), and MAP (*n* = 43) in comparison with controls (*n* = 32). We discovered that persistent MA use had a robust immunosuppressive impact on all immunological profiles. The most reliable biomarker profile of MA use is the combination of substantial CIRS suppression and a rise in selected pro-inflammatory cytokines, namely CCL27 (CTACK), CCL11 (eotaxin), and interleukin (IL)-1α. In addition, MA dependency is associated with increased immunosuppression, as demonstrated by lower stem cell factor levels and higher IL-10 levels. MAP is related to a significant decrease in all immunological profiles, particularly CIRS, and an increase in CCL5 (RANTES), IL-1α, and IL-12p70 signaling. In conclusion, long-term MA use and dependency severely undermine immune homeostasis, whereas MAP may be the consequence of increased IL-1α – CCL5 signaling superimposed on strongly depleted CIRS and Th-1 functions. The widespread immunosuppression established in longstanding MA use may increase the likelihood of infectious and immune illness or exacerbate disorders such as hepatitis and AIDS. Furthermore, elevated levels of CCL5, CCL11, CCL27, IL-1α, and/or IL-12p70 may play a role in the peripheral (atherosclerosis, cutaneous inflammation, immune aberrations, hypospermatogenesis) and central (neuroinflammation, neurotoxic, neurodegenerative, depression, anxiety, and psychosis) side effects of MA use.

## Introduction

Methamphetamine (MA), a psychostimulant, is the second most frequently used illegal substance worldwide [1, 2]. Between 2009 and 2018, the number of people who used MA increased from 210 million to 265 million worldwide [3]. In Southeast Asia, particularly Thailand, one of the most popular drugs is MA speed tablets, which combine MA and caffeine [4, 5]. In recent years, crystal meth (ice), a crystalline and pure version of MA, has gained popularity and was introduced as a chemsex substance, including among those who inject drugs [6, 7].

An increase in dopamine, norepinephrine, and serotonin turnover causes the stimulant effects of MA on the central nervous system [8]. In addition, the use of MA causes significant neurological and physical consequences, such as sleep disturbances, aggressive behavior, anxiety, sadness, depression, and psychosis, including delusions and hallucinations [1, 9]. MA-induced psychosis (MAP) is characterized by persecuting delusions, hallucinations, and conceptual disorganization [10–12]. Around 3–23% of individuals who frequently use MA in a population sample with MA use reported MAP or psychotic symptoms [13]. Around 40% of people with MA dependency in a hospital-based research group may have psychotic symptoms, such as paranoia, throughout their lifetime use of MA [14]. MAP occurs over the course of MA use, including acute intoxication and chronic use [15]. Typically, MAP may be present for a brief period, a few days to one month after the cessation of MA use; nevertheless, 38.8% of patients have a continuous course of psychosis, suggesting an association between MAP and primary psychotic disorders, such as schizophrenia [16].

A number of studies demonstrated that MA use may disrupt immunological homeostasis and suppress immune functions [17], including suppressing the production of T-helper (Th)-1 cytokines [18], T-cell proliferation [19], and the number of lymphocytes and immune cells [20, 21]. Nitrostyrene, an amphetamine derivative, decreases interleukin (IL)-12 and IL-6 concentrations [22]. Earlier research showed that MAP is associated with symptoms of immunological activation and inflammation, as well as neuroinflammation, as evidenced by elevated IL-6 and IL-8 [23], as well as elevated oxidative stress and decreased antioxidant defenses [12]. MA may affect antigen-presenting cells (APCs) in the brain, leading to an increase in pro-inflammatory cytokines such as IL-1β, IL-6, IL-8, interferons (IFN), and tumor necrosis factor (TNF)-α [24]. A few studies have reported changes in immunological profiles in individuals who use MA or have MA dependence [25–27]. Nevertheless, the immune profile of MAP has remained elusive.

MAP is often used and investigated as a model of schizophrenia because of its overlapping symptoms and biomarker characteristics, such as elevated dopamine turnover [28]. There is now evidence that schizophrenia, and particularly its more severe phenotype deficit schizophrenia, is a neuro-immune and neuro-oxidative disorder [29–31], with elevated levels of pro-inflammatory cytokines such as IL-1β, IL-6, IL-12p70, and TNF-α [32, 33], activation of macrophage M1, Th-1, Th-2, Th-17, and T regulatory (Treg) phenotypes, as well as activation of the immune-inflammatory response system (IRS) and the compensatory immune-regulatory systems (CIRS), which tend to downregulate the IRS and prevent hyperinflammation [31]. However, no research has studied the comprehensive immunological profiles of MA use, MA dependence, and MAP, including M1, Th-1, Th-2, Th-17, IRS, CIRS, chemokine, and growth factor profiles, to determine whether these conditions are characterized by immune activation or suppression.

Hence, we explored peripheral blood immune profiles in MA use, MA dependence, and MAP as compared with controls to examine whether these conditions are accompanied by immune activation or immunosuppression and which immune profiles and cytokines are specifically involved in the three conditions. More specifically, the a priori hypothesis was to detect increased levels of pro-inflammatory cytokines such as IL-1β, IL-6, IL-8, IFN-γ, and TNF-α in MAP, and a moderate immunosuppression in MA dependency.

## Methods

### Participants

This research recruited 173 participants, 32 controls, and 141 patients with MA use from the Princess Mother National Institute on Drug Abuse Treatment (PMNIDAT), Thailand, including those with no MA dependency (*n* = 51), MA dependence (*n* = 47), and MAP (*n* = 43). A verbal notice was made at King Chulalongkorn Memorial Hospital to recruit controls (*n* = 32) who had never taken MA in their lives and did not have any drug dependency except for a tobacco use disorder. By age and gender, the controls were matched with the MA group. All participants were between the ages of 18 and 65. Participants with severe neuropsychiatric disorders, such as schizophrenia, bipolar disorder, psycho-organic disorders, and schizo-affective disorder, as well as subjects with (auto)immune disorders, such as psoriasis, inflammatory bowel disease, rheumatoid arthritis, and lupus erythematosus, were excluded from the study. We also omitted women who were pregnant or nursing. The data were collected between March - August 2022, and the project was approved by the Institutional Review Board of the Faculty of Medicine of Chulalongkorn University and the PMNIDAT (No. 28/2565).

### Clinical assessment

Using the Thai version of the Semi-Structured Assessment of Drug Dependency and Alcoholism (SSADDA), demographics, drug use data including tobacco, alcohol, cannabis, and MA, and diagnoses were acquired [14, 34]. Based on the Fourth Version of the Diagnostic and Statistical Manual of Mental Disorders (DSM-IV), the SSADDA is a thorough interview to identify drug dependency and associated mental illnesses [35]. The MA dependency group comprised patients who complied with 6-7 criteria on the DSM-IV MA dependent scale (utilizing the SSADDA), whereas patients with 0-1 criteria were recruited for the study group with MA use. Persons having a history of psychotic symptoms while taking MA, including hallucinations and/or delusions, were selected for the MAP group if they answered “yes” to any of the psychosis section of the SSADDA questions (i.e., probing code = yes, always due to drugs (MA)). Using the SSADDA, we also determined the length of heavy MA use (in months), the daily quantity of MA (in milligrams; mg), and the forms of MA, such as crystal meth, speed pills, or both. We also evaluated lifetime and present alcohol and illicit substance use. We estimated the maximum number of alcoholic drinks per day; one standard alcoholic drink is equivalent to 10 g of pure alcohol. The interviewers were two psychologists with more than five years of training and experience in performing SSADDA interviews. Before finishing the interview data, all interviews were cross-checked by a second interviewer.

### Measurement of cytokines

Early in the morning (between 8:00 and 9:00 a.m.), 5 ml of fasting venous blood was drawn from each participant, using a disposable syringe. Blood was stored at −80 °C until thawed for biomarker assays. The samples were placed at room temperature for 1 h, then aliquots 25 µl. whole blood were diluted 1:4 (sample: diluent) after that, the standard dilutions were prepared. The concentrations of 48 cytokines, chemokines, and growth factors were measured in duplicate using Bio-Plex Multiplex Immunoassays (Bio-Rad Laboratories Inc., USA). 50 µl of the diluted samples were combined with 50 µl of the microparticle cocktail (containing cytokines/chemokines per well of a 96-well plate) and incubated for 1 h at room temperature while shaking at 850 rpm. The plates were rinsed three times, then 50 µl of diluted Streptavidin-PE was added to each well, and it was incubated for 10 min at room temperature. 125 µl of assay buffer was added, and the wells were shaken for 30 s at room temperature (850 rpm) prior to being read with the Bio-Plex® 200 System (Bio-Rad Laboratories, Inc.). In the present study, we employed the concentrations of the different cytokines/chemokine/growth factors in the statistical analyses. Concentrations that were not detectable (i.e., the lower limit out of range values) were censored and we imputed these censored data with the sensitivity values of each assay. Cytokines/chemokines/growth factors with concentration levels that were >45% out of range were excluded from the analysis concerning the solitary analytes. Nevertheless, we also computed different immune profiles (see Introduction), including M1, Th-1, Th-2, IRS, and CIRS profiles, and all analytes (even those with <45% detectable concentrations) may be used toward that end [30, 36], except those with <7% detectable concentrations (GM-CSF, IL-2, IL-3, IL-5, IL-15, and β-NGF). The reason is that even a smaller number of measurable concentrations (> more than 12) may contribute to the immune profiles. Electronic Supplementary File (ESF), Table 1 lists all cytokine/chemokines/growth factors measured here, as well as their names, gene IDs and % measurable analytes (> lower limit of out-of-range concentration). ESF, Table 2 shows how we constructed M1, Th-1, Th-2, IRS, and CIRS immune profiles, based on our previous publications [30, 36, 37] Unfortunately, we were unable to construct the Th-17 profile because its major players, IL-6 and IL-17, were not measurable in many blood samples. We also computed the ratio z transformation of IL-12p70 - z IL-12p40, because IL-12 signaling via IL-12p70 may be inhibited via IL-12p40 [38].

**Table 1:** Demographic and clinical data in controls (C), patients with methamphetamine (MA)-use, MA-dependence, and MA-induced psychosis (MAP).

Variables	C^A^ *n* = 32	MA use^B^ *n* = 51	MA dependence^C^ *n* = 47	MAP^D^ *n* = 43	F/X^2^	df	*p*
*Demographics*							
Female/male	16/16	25/26	23/24	18/25	0.71	3	0.870
Age (years)	31.8 (10.7)	33.6 (6.8)^C^	29.0 (8.1)^B^	31.0 (7.3)	2.75	3/169	0.044
Marital status (S/M/D)	25/7/0	40/8/3	36/4/7	27/2/14	FFHET	–	<0.001
Body mass index (kg/m^2^)	26.2 (5.8)	21.5 (3.8)	22.4 (4.4)	25.1 (4.9)	9.40	3/169	<0.001
*MA use variables*							
Duration of heaviest use (months)		8.8 (30.3)^C^	84.9 (87.9)^B,D^	25.7 (47.1)^C^	21.32	2/138	<0.001
Daily amount of heaviest use (mg)		106.3 (113.9)^C,D^	324.3 (343.7)^B,D^	906.9 (908.0)^B,C^	26.51	2/138	<0.001
DSM-IV criteria number^a^	0.0^B,C,D^	0.3 (0.5)^A,C,D^	6.7 (0.7)^A,B^	6.9 (0.4)^A,B^	KWT	–	<0.001
MA dependence severity (z score)	−0.986 (0.0)^C,D^	−0.801 (0.270)^C,D^	0.770 (0.688)^A,B^	0.825 (0.780)^A,B^	KWT	–	<0.001
Heaviest MA use (N/Cr/SP/Both)	32/0/0/0	0/22/29/0	0/12/30/5	0/20/13/10	–	–	–
*Alcohol use variables*							
Maximum alcohol drinks per day^b^	5.8 (6.7)^B,C,D^	9.3 (13.6)^A,D^	13.7 (12.2)^A,D^	21.3 (24.9)^A,B,C^	6.95	3/169	<0.001
Severity of alcohol use (z scores)	−0.336 (0.778)^C^	−0.243 (0.875)^C^	−0.007 (0.929)^C^	0.546 (1.1541)^A,B,C^	7.17	3/170	<0.001
Lifetime alcohol dependence (Y/N)	0/31	2/46	8/37	21/21	40.93	3	<0.001
Current alcohol drinking (Y/N)	2/29	2/46	1/44	0/43	2.92	3	0.404
*Tobacco use variables*							
Lifetime tobacco use (Y/N)	8/24	43/8	43/4	41/1	69.62	3	<0.001
Current tobacco smoking (Y/N)	8/24	15/36	12/35	4/39	6.00	3	0.112
*Cannabis use variables*							
Lifetime cannabis use (Y/N)	7/25	18/33	36/11	32/11	37.32	3	<0.001
Current cannabis use (Y/N)	0/32	0/51	2/45	0/43	FFHET	–	0.168

All data are shown as mean (SD), analyzed using analysis of variance (F) or Kruskal Wallis test (KWT); or as ratios, analyzed using χ^2^ tests or Fisher–Freeman–Halton exact test (FFHET); ^A,B,C,D^: protected post-hoc comparisons among group means, *S/M/D* single/married/divorced, *N/Cr/SP/Both* none/crystal MA/speed pills/both, *Y/N* yes/no.

^a^Diagnostic and Statistical Manual on Mental Disorders (DSM-IV) symptom count or number of criteria for methamphetamine dependence.

^b^Alcohol standard drink or unit (1 drink = 10 g of pure alcohol).

**Table 2:** Measurements of immune profiles in controls (C), patients with MA-use, MA-dependence, and MA-induced psychosis (MAP).

Z scores	C^A^ *n* = 32	MA use^B^ *n* = 51	MA dependence^C^ *n* = 47	MAP^D^ *n* = 43	F	*P*-values
M1	0.776 (0.146)^B,C,D^	0.236 (0.117)^A,D^	0.113 (0.118)^A,D^	−0.971 (0.125)^A,B,C^	31.87	<0.001
Th-1	0.912 (0.139)^B,C,D^	0.081 (0.111)^A,D^	0.251 (0.112)^A,D^	−1.048 (0.118)^A,B,C^	43.79	<0.001
IL-12_70_ - IL-12_40_	−0.740 (0.155)^B,C,D^	−0.307 (0.124)^A,C,D^	0.173 (0.125)^A,B,D^	0.711 (0.132)^A,B,C^	20.34	<0.001
Th-2	0.919 (0.144)^B,C,D^	0.149 (0.115)^A,D^	0.119 (0.116)^A,D^	−0.985 (0.123)^A,B,C^	37.00	<0.001
IRS	0.609 (0.159)^B,D^	0.136 (0.127)^A,D^	0.211 (0.128)^D^	−0.836 (0.135)^A,B,C^	19.34	<0.001
CIRS	1.010 (0.135)^B,C,D^	0.085 (0.108)^A,D^	0.181 (0.109)^A,D^	−1.042 (0.115)^A,B,C^	48.65	<0.001
IRS/CIRS	−0.693 (0.175)^B,C,D^	0.088 (0.140)^A^	0.053 (0.141)^A^	0.356 (0.149)^A^	7.42	<0.001
Chemokines	0.760 (0.141)^B,C,D^	0.373 (0.113)^A,C,D^	0.014 (0.114)^A,B,D^	−1.015 (0.120)^A,B,C^	37.51	<0.001
Growth factors	0.805 (0.144)^C,D^	0.448 (0.115) ^C,D^	−0.166 (0.116)^A,B,D^	−0.957 (0.122)^A,B,C^	37.00	<0.001
PC_immune	0.863 (0.140)^B,C,D^	0.202 (0.112) ^A,D^	0.151 (0.113)^A,D^	−1.042 (0.119)^A,B,C^	40.69	<0.001

All results of univariate GLM analyses; shown are the estimated marginal mean (SE) values (in z scores) after covarying for body mass index, sex, age, current smoking, and current alcohol use (all df = 3/164). ^A,B,C,D^: protected post-hoc comparisons among group means.

*M1* macrophage M1, *Th* T helper, *IL* interleukin, *IRS* immune-inflammatory responses system, *CIRS* compensatory immune-regulatory system, *PC_immune* first principal component extracted from the immune profiles.

### Statistical analysis

Using Pearson’s product-moment correlation coefficients, correlations between variables were analyzed. The analysis of contingency tables was used to compare variables depending on their categories (Chi-square tests). Analysis of variance was employed to examine the differences in continuous variables across the 4 study groups (controls (C), MA use, MA dependence, and MAP). We used univariate generalized linear models (considering the impacts of gender, age, BMI, current tobacco use, and alcohol use disorder) to examine the associations among these groups and cytokines and immune profiles. The Levene test for equality of variances was checked to ascertain that the variances of the study samples are equal. At a significance level of *p* < 0.05, pairwise comparisons of group means were conducted to detect differences between the four study groups. We did not use p-corrections for false discovery rate, because it is well established that the cytokines/chemokines/growth factors measured here act in a tight network and show strongly integrated responses [30]. Binary logistic regression analysis was performed to delineate the most important predictors of MAP versus MA use + MA dependence, and MA dependence versus MA use. Using the overfit criterion as entry and/or removal criterion (maximum effects number set at 5) we performed forward stepwise automatic linear modeling analyses (allowing for the confounders) and consequently examined the model using a manual regression analysis. We checked the residual distributions of the final model, the variance inflation factor and tolerance to identify any collinearity or multicollinearity concerns, and heteroskedasticity using the White and modified Breusch-Pagan homoscedasticity tests. We calculated the partial regression analysis of immune data on clinical data, model statistics (F, df, and *p* values, total variance explained, which was used as effect size), and the standardized β coefficients, t statistics (with exact *p*-value) for each predictor. The significance threshold of all statistical analyses was calculated using two-tailed tests with a value of 0.05. Where needed, the cytokines/chemokines/growth factors were introduced in the statistical analyses after transformations, including log10, fractional rank-based normal transformations, and Winsorization. We used principal component analysis (PCA) as a feature reduction method, and the first PC was considered adequate when the variance explained (VE) in the variables was >50%, and all have a loading >0.66, the factoriability of the correlation matrix using the Kaiser-Meyer-Olkin (KMO) Measure of Sampling Adequacy, the Bartlett’s sphericity and the anti-image correlation matrix were accurate. All the statistical studies were conducted using IBM, SPSS windows version 28. A priori sample size calculation was conducted using G*Power 3.1.9.4; for an analysis of covariance with 5 covariates, effect size of 0.27, alpha = 0.05 (two tailed), power = 0.8, and 4 groups the sample size should be at least 154.

## Results

### Demographics and clinical data in the four study groups

To examine differences in demographic data between the study groups we used analysis of variance and analysis of contingency tables. Table 1 shows the sociodemographic and clinical data of the 4 study groups. There were no significant differences in the sex ratio among groups, although there were some differences in age, BMI, and marital status between the groups. Duration of heaviest MA use was significantly greater in MA dependence than in MAP and MA use. The MA daily amount of heaviest MA use was significantly different between the three groups are increased from MA use to MA dependence to MAP. The DSM criteria number and MA dependence severity were significantly higher in the MAP and MA dependence groups than in the MA use group. The severity of MA dependence and the DSM-IV number of dependence criteria were significantly higher in MA dependence and MAP than in those with MA use. There were significant differences in lifetime, but not current, tobacco and cannabis use among the four study groups.

### Lowered immune profiles in MA use, MA dependence, and MAP

We performed PCA, which is a feature reduction method, to reduce the various immune profiles into one construct, which reflects a generalized index of immune function. Using PCA, we were able to extract one PC from M1, Th-1, Th-2, IRS, CIRS, chemokine and growth factor profiles (VE = 84.7%, all loadings >0.878, KMO = 0.896, Bartlett’s test: χ^2^ = 1652.808, df = 21, *p* < 0.001), labeled PC_immune. The latter is, therefore, an overall index of immune functions. These results further show that no p-correction for false discovery rate may be used as all immune responses are strongly interrelated [30].

To examine differences in the immune profiles between the study groups we used analysis of variance. Table 2 shows the immune profiles of the four study groups. All the cytokine profiles, including PC_immune, were significantly different between the study groups (*p* < 0.001). The MAP group had the lowest M1, Th-1, Th-2, IRS, CIRS, chemokine, growth factor, and PC_immune scores but higher IL12_70_ - IL20_40_ scores as compared with the three other groups. In addition, MA dependence and/or MA use had lower z-scores of M1, Th-1, Th-2, IRS, CIRS, chemokine, growth factor, and PC_immune scores than controls. The PC_immune score was lower in MAP than in all other subjects. The IL12_70_ - IL20_40_ scores were higher in patients than controls. A post-hoc analysis to compute the achieved power for the least significant comparison in Table 2 (IRS/CIRS), showed that the achieved power (at *α* = 0.05, 4 groups, 5 covariates, *n* = 174) was 0.987. The achieved power for all other comparisons was 1.0. ESF, Figures S1–S36 show the levels of the measurable cytokines/chemokines/growth factors in controls, MA-use, MA dependence, and MAP.

All in all, the results show significant differences in immune profiles between patients who use MA and controls, and significant decreases in the generalized immune function index in patients who use MA and especially in those with MA.

### Intercorrelation matrix

To examine the associations between MA use features and immune profiles we computed the correlation matrix (based on Pearson’s product moment correlation coefficients) among both sets of data. Table 3 shows the associations between MA use and dependence features and immune profiles. The MA dose was significantly and negatively correlated with the M1, Th-1, Th-2, IRS, CIRS, chemokine, growth factor, and PC_immune profiles, and positively with IL12_70_ - IL20_40_ and the IRS/CIRS ratio. The duration of MA use showed an inverse association with the growth factor profile. The severity of MA dependence was significantly associated with M1, Th-1, Th-2, IRS, CIRS, chemokine, growth factor, and PC_immune profiles, with a positive correlation with IL12_70_ - IL20_40_ and the IRS/CIRS ratio.

**Table 3:** Intercorrelation matrix between methamphetamine (MA) use and dependence features and immune profiles.

Variables	MA amount of use^a^	MA duration of use^b^	MA dependence severity^c^
M1	−0.338 (<0.001)	−0.083 (0.274)	−0.438 (<0.001)
Th-1	−0.396 (<0.001)	−0.044 (0.560)	−0.402 (<0.001)
IL12_70_ - IL12_40_	0.321 (<0.001)	0.138 (0.070)	0.475 (<0.001)
Th-2	−0.376 (<0.001)	−0.062 (0.419)	−0.430 (<0.001)
IRS	−0.280 (<0.001)	−0.046 (0.548)	−0.321 (<0.001)
CIRS	−0.378 (<0.001)	−0.089 (0.243)	−0.436 (<0.001)
IRS/CIRS	0.169 (0.026)	0.074 (0.330)	0.199 (0.009)
Chemokines	−0.368 (<0.001)	−0.102 (0.182)	−0.508 (<0.001)
Growth factors	−0.382 (<0.001)	−0.175 (0.021)	−0.557 (<0.001)
PC_immune	−0.381 (<0.001)	−0.074 (0.331)	−0.449 (<0.001)

Shown are Pearson’s product moment correlation coefficients (with exact *p* value, all: *n* = 174).

*M1* macrophage M1, *Th* T helper, *IL* interleukin, *IRS* immune-inflammatory responses system, *CIRS* compensatory immune-regulatory system, *PC_immune* first principal component extracted from the immune profiles.

^a^Daily amount of heaviest use of MA.

^b^Duration of heaviest use of MA.

^c^z unit-weighted composite score based on daily amount of heaviest use of MA, duration of heaviest use of MA and the Diagnostic and Statistical Manual on Mental Disorders (DSM-IV) number of criteria for MA dependence.

All in all, the results show that MA amount of use and dependency severity are associated with all immune profiles, including the generalized immune function index.

### The general immune profile is predicted by MA dose and dependency criteria

**Fig. 1:**
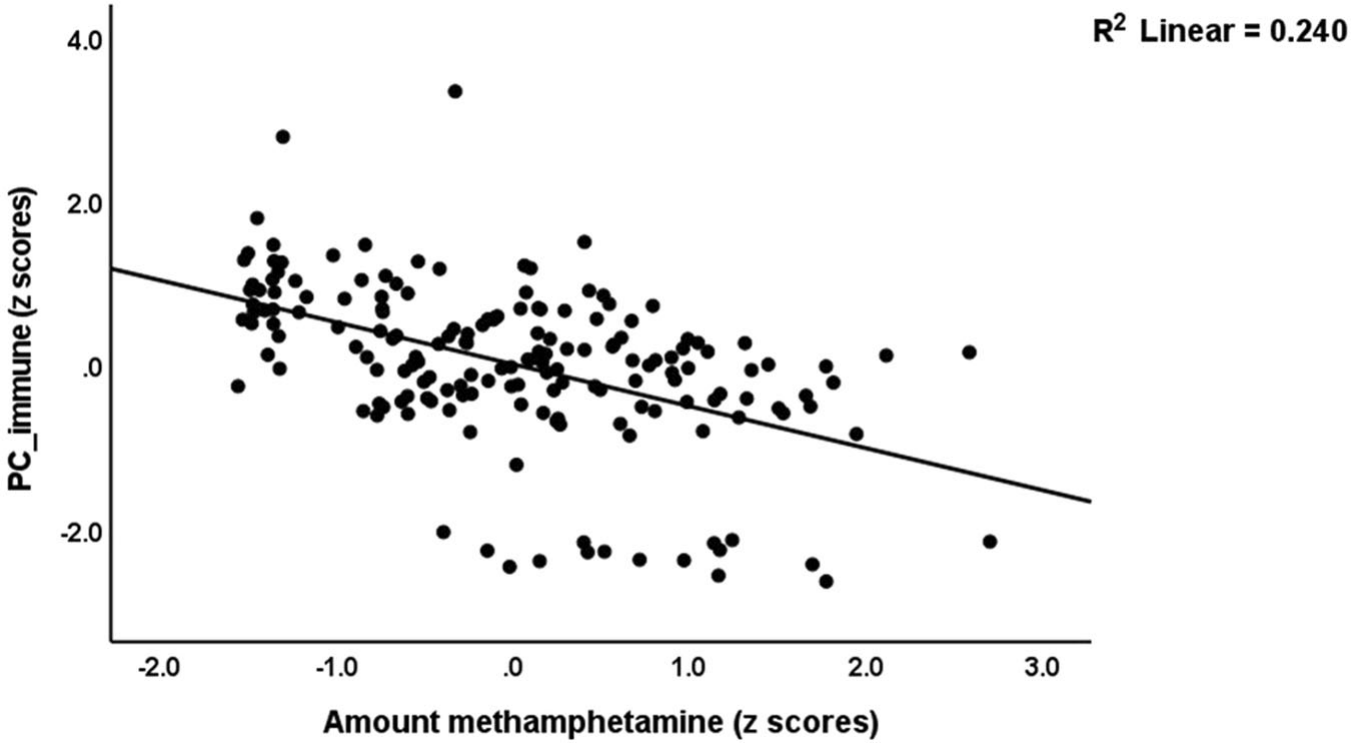
Partial regression of a generalized index of immune function (PC_immune) on the amount of methamphetamine.

To examine the associations between PC_immune and important clinical data, including MA dose and dependency criteria, we used multiple regression analysis with PC_immune as the dependent variable and the clinical data as explanatory (independent) variables). Table 4 shows two different regression models predicting PC_immune. The first model was performed using MA features only and shows that PC_immune is significantly and inversely predicted by the number of dependency criteria and MA dose. Figure 1 shows the partial regression of PC_immune on MA dose. In addition, we checked whether the association between PC_immune and the 4 diagnostic groups (controls and the three MA groups) was affected by alcohol dependence or cannabis features. Regression #2 shows that PC_immune is associated with the 4 diagnostic groups and alcohol dependence, indicating that the groups and alcohol dependence have independent effects. Cannabis features did not have any effects on PC_immune.

**Table 4:** Results of multiple regression analysis with the immune profile (PC_immune) as dependent variable.

		Parameter estimates with statistics			Model statistics and effect size			
Dependent variable	Explanatory variables	ß	t	*p*	R^2^	F	df	*p*
#1. PC_immune	Model				0.268 – –	31.11 – –	2/170	<0.001 – –
	Number of criteria^a^	−0.189	−2.03	0.044				
	MA dose^b^	−0.366	−3.93	<0.001				
#2. PC_immune	Model				0.401 – –	54.56 – –	2/163	<0.001 – –
	C/MAU/MAD/MAP^c^	−0.743	−10.60	<0.001				
	Alcohol dependence	0.198	2.94	0.004				

PC_immune: first principal component extracted from macrophage M1; Th-1 (T-helper), Th-2, immune-inflammatory responses system; compensatory immune-regulatory system, growth factors, and chemokines.

*MA* methamphetamine.

^a^Diagnostic and Statistical Manual on Mental Disorders (DSM-IV) number of criteria for MA dependence.

^b^Daily amount of heaviest use of MA.

^c^Duration of heaviest use of MA; ordinal variable with C: controls = 0, MAU: MA use = 1, MAD: MA dependence = 2; and MAP: MA-induced psychosis = 3.

All in all, we found that the number of dependency criteria and MA dose were associated with suppressed immune functions.

### MAP and MA dependence are predicted by immune data

To delineate the most important immune profiles or cytokine/chemokines/growth factors that are associated with MA use, MA dependence, and MAP, we performed binary regression analyses with these groups (present versus not present) as dependent variables and the immune data as explanatory variables (while allowing for the effects of age, sex, etc). Table 5 shows the results of these logistic binary regression analyses with MAP, MA dependence, and MA use as dependent variables. Binary logistic regression #1 shows that MAP (reference group = MA use + MA dependence) is associated with lowered CIRS, increased MA dose, number of DSM-IV MA dependence criteria, and alcohol dependence (χ^2^ = 60.69, df = 2, *p* < 0.001, correctly classified subjects: 82.5%, Nagelkerke = 0.492). Regression #2 shows that MAP (reference group = MA use + MA dependence) was strongly associated with Th-1 and CIRS profiles (inversely) and CCL5 and IL-1α (positively) (χ^2^ = 96.41, df = 4, *p* < 0.001, correctly classified subjects: 87.3%, Nagelkerke=0.698). Regression #3 shows an alternative prediction of MAP and that CIRS (inversely), IL12_70_ - IL20_40_ and IL-1α (positively) are significant explanatory variables (χ^2^ = 103.4, df = 3, *p* < 0.001, correctly classified cases: 86.7%, Nagelkerke = 0.667). Regression #4 examined the prediction of MAP versus all other patients and controls and found that the same variables as shown in regression #2 predicted MAP (χ^2^ = 116.812, df = 4, *p* < 0.001, correctly classified cases: 89.8%, Nagelkerke = 0.726). Regression #5 shows that IRS/CIRS was the single best predictor of MAP (versus MA dependence) (positive correlation). Binary logistic regression #6 examines the prediction of MA dependence (MA use as reference group) and shows that MA dependence was associated with SCF (inversely) and IL-10 (positively) (χ^2^ = 107.362, df = 2, *p* < 0.001, correctly classified cases: 94.1%, Nagelkerke = 0.888). The last regression (#7) shows that the best discrimination of MA patients versus controls was obtained using CIRS (inversely) and CTACK + CCL11 + IL-1α (positively) as predictors (χ^2^ = 92.793, df = 2, *p* < 0.001, correctly classified cases: 93.1%, Nagelkerke = 0.672).

**Table 5:** Results of logistic binary regression analysis with methamphetamine (MA)-induced psychosis (MAP), MA dependence (MAD), or MA use (MAU) as dependent variables and immune profiles or single cytokines as explanatory variables.

Dependent variable (reference group)	Explanatory variables	B	SE	Wald	df	*p*	OR	95% CI
#1. MAP (MAU + MAD)	CIRS	−2.611	0.749	12.17	1	<0.001	0.07	0.02; 0.32
	MA dose	0.677	0.312	4.70	1	0.030	1.97	1.07; 3.63
	DSM-IV MA criteria	2.803	1.348	4.33	1	0.038	16.50	1.18; 2.31
	Alcohol dependence	0.340	0.146	5.46	1	0.019	1.41	1.06; 1.87
#2. MAP (MAU + MAD)	Th-1	−2.904	0.990	8.60	1	0.003	0.06	0.01; 0.38
	CIRS	−2.100	1.008	4.34	1	0.037	0.12	0.02; 0.88
	CCL5	1.241	0.468	7.14	1	0.008	3.46	1.39; 8.60
	IL-1$\propto$	1.869	0.600	9.71	1	0.002	6.48	2.00; 20.99
#3. MAP (MAU + MAD)	IL-12_70_-IL12_40_	0.549	0.279	3.91	1	0.047	1.73	1.01; 2.98
	CIRS	−3.330	0.633	27.66	1	<0.001	0.036	0.01; 0.12
	IL-1α	1.784	0.533	11.23	1	<0.001	5.96	2.10; 16.91
#4. MAP (controls+ MAU + MAD)	CIRS	−2.127	1.001	4.52	1	<0.034	0.12	0.02; 0.85
	Th-1	−2.901	0.991	8.57	1	0.003	0.06	0.008; 0.38
	CCL5	1.249	0.463	7.28	1	0.007	3.49	1.41; 8.65
	IL-1$\propto$	1.887	0.598	9.97	1	0.002	6.60	2.05; 21.29
#5. MAP (MAD)	IRS/CIRS	0.501	0.228	4.81	1	0.028	1.65	1.05; 2.58
#6. MAD (MAU)	SCF	−2.90	1.057	7.50	1	0.006	0.06	0.01; 0.44
	IL-10	8.70	2388	13.28	1	<0.001	5998	5568; 64620
#7. MAU (controls)	CIRS	−4.121	0.730	31.85	1	<0.001	0.02	0.004; 0.07
	CCL27 + CCL11 + IL-1α	2.679	0.705	14.44	1	<0.001	14.58	3.66; 58.05

*M1* macrophage M1, *Th* T helper, *IL* interleukin, *IRS* immune-inflammatory responses system, *CIRS* compensatory immune-regulatory system, *PC_immune* first principal component extracted from the immune profiles, *SCF* stem cell factor, *CCL27 or CTACK* cutaneous T-cell attracting chemokine.

All in all, the results show that MA use, MA dependence and MAP are predicted by different immune profiles and selected cytokines.

## Discussion

### Immune profiles in MA use and dependence

The first major finding is that chronic MA use reduces the first factor extracted from all immunological profiles (M1, Th-1, Th-2, IRS, CIRS, chemokine, and growth factors), indicating widespread immunosuppression. These findings expand upon those of recent papers demonstrating the many immunosuppressive effects of MA. MA at pharmacological doses exerts immunosuppressive effects on antigen-presenting cells, including macrophages and dendritic cells, reduces T cell proliferative activity, and inhibits receptor-mediated phagocytosis of antibody particles and MHC class II antigen presentation by T cells [19, 39]. In addition, MA treatment of splenocytes has a substantial influence on antigen-induced proliferation and macrophage phagocytosis [40]. Two weeks of MA treatment inhibits stimulated lymphoproliferative responses in mice [20]. In rodent models, injection of MA reduces the number of T lymphocytes in the spleen, including CD4 and CD8 cells, and the number of macrophages, dendritic cells, and natural killer cells [21]. Furthermore, treatment of MA lowers the production of Th-1 cytokines, including IFN-γ, and IL-2, but has no effect on the production of IL-6 and IL-4 [18].

Importantly, the immunosuppression in individuals with MA use is strongly associated with increasing doses of MA (but not the duration of the heaviest MA use) and with the severity of MA dependence. This indicates that MA has a strong suppressant effect on IRS and CIRS products. Overall, our data on M1 and Th-1 profiles are consistent with animal studies showing decreased M1 and Th-1 activity following MA exposure. Despite this, our findings indicate that chronic MA use additionally suppresses Th-2 and CIRS profiles, as well as the chemokine and growth factor subnetworks. This is important, as the latter subnetwork is intimately connected with the cytokine-chemokine network [41]. In addition, we discovered that prolonged MA use lowers the levels of several cytokines/chemokines/growth factors, such as FGF, GRO, IL-1β, sIL-1RA, sIL-2R, IL-4, IL-8, IL-12p40, IL-13, IL-18, LIF, CCL7 (MCP3), CSF, MIF, CXCL9 (MIG), SCF, and TRAIL.

Obviously, such a general decrease in immune processes may be accompanied by dysfunctions in host immunity, as observed in persons who used MA, resulting in greater susceptibility to acquire new infections and a worsening of infectious diseases, including hepatitis and AIDS [42].

### Factors explaining MA-induced immunosuppression

Several variables may account for the significant immunosuppression generated by persistent MA use. First, MA may interfere with the cell cycle machinery, thereby preventing T cell proliferation and a proper adaptive immune response [19]. MA may reduce T cell cycle gene expression, including cyclin E, CDK2, and E2F, resulting in a prolonged G1/S phase transition [19]. Second, injection of MA causes apoptosis in macrophages and T cells [43, 44]. Third, MA-stimulated catecholamine turnover may suppress the production of IL-6, TNF-α, and sIL-1RA, therefore influencing the IRS and CIRS [45]. Fourth, exposure to MA, a weak base, may interfere with the pH maintenance of the more acidic organelles, which govern protein breakdown and surface receptor expression [39, 40]. The effects of MA on the pH gradient may impair acidic organelles inside immune cells, MHC class II antigen processing, antigen presentation by dendritic cells to T cells, and ultimately the immunological response [39, 40]. Fifth, MA exposure may induce T cell mRNA expression of the trace amine-associated receptor 1 (TAAR1) [46], which is involved in rheostasis, homeostasis, and cAMP signaling and interacts with monoamine turnover [47, 48]. This indicates that MA-induced TAAR1 expression may downregulate Th-1-like cytokines such as IL-2 [46].

### Signs of immune activation in MA use and dependence

Our second major observation is that persistent MA use is also associated with indicators of immunological activation. Various analytes were significantly elevated, such as CCL27 (CTACK) in subjects who used MA, and IL-1α, IL-12_p70_, and CCL5 (RANTES) in subjects with MA dependence. In addition, not all analytes were lowered in persons who used MA, including CCL11 (eotaxin), IL-9, IL-16, CXCL10 (IP10), CCL3 (MIP1α), CCL4 (MIP1β), TNF-α and TNF-β (in both MA use and dependence), and G-CSF, HGF, CCL2 (MCP1), PDGF-BB, CXCL12 (SDF-1α), IL-9, and IL-10 (in MA dependence). These findings expand on the findings of prior investigations. For example, administration of MA to microglia markedly elevates IL-12_p70_ [49], and MA exposure substantially increases IL-12 levels in the kidney and liver of mice [50]. Moreover, MA induces T cell proliferation in the brain via upregulating IL-15 in astrocytes [51]. Macaques afflicted with Simian Immunodeficiency Virus may produce more CCL5 if they are treated with MA [52]. MA exposure elevates TNF-α levels in selected mouse brain areas, which are partially mediated by redox processes [53]. Another research paper showed that MA exposure may considerably boost TNF-α production even in the presence of Th-1 suppression [18].

Considering that people who use MA have a suppressed immune system, the significant or relative increase in some cytokines/growth factors may be relevant. This is further supported by a higher IRS/CIRS ratio in individuals who use MA compared to controls. Hence, despite the overall immunosuppression, there is a relative rise in pro-inflammatory cytokines/chemokines, as well as growth factors (G-CSF, PDGF-BB, and CXCL12 or SDF-1α) that may fuel the synthesis of these cytokines/chemokines. In fact, a combination of a decreased CIRS profile and an increase in pro-inflammatory cytokines/chemokines (CCL27 + CCL11 + IL-1α) is the best biomarker profile of MA use. It is noteworthy to note that CD4+ cells, which are inhibited during MA use, also exhibit indicators of activation, such as increased expression of CD150 and CD226 [21].

All in all, not only generalized immunosuppression but also signs of immune activation are important to understand the pathophysiology of MA use. For example, CCL27 (CTACK or cutaneous T cell-attracting chemokine) is a cutaneous chemokine that attracts lymphocyte-associated antigen (CLA)+ memory cells, which are implicated in cutaneous inflammatory lesions [54]. As such, increased CCL27 may perhaps play a role in MA-associated skin damage, including itching (due to meth mites), lesions, excoriations, and ulcers [55]. IL-1α, IL-12_p70_, and CCL5 are pro-inflammatory cytokines/chemokines that may contribute to the inflammatory pathways and the accompanying brain dysfunctions [42] as well as peripheral inflammation-linked disorders, including cardiovascular diseases, which frequently occur in persons who use MA [12]. CCL11 levels may be associated with greater anxiety, depression, and cognitive deficits among individuals who use MA [26].

Moreover, the transition from moderate MA use to severe MA dependency (greater dosage and longer duration of MA use) is characterized by decreased SCF (stem cell factor) and increased IL-10 levels. SCF is a cytokine that plays a major role in hematopoiesis and spermatogenesis; consequently, decreased SCF may contribute to the decreased lymphoproliferative responses to MA (see discussion above) and the decreased sperm count, motility, and morphology in persons who use MA [56]. In response to MA exposure, IL-10 levels are elevated in the plasma of both mice and humans [50, 57]. Such a non-protective immunological response to IL-10 may restrict T cell proliferation even further.

### The immune profile of MAP

The third major finding of the study is that MAP is characterized by a) extremely reduced levels of M1, Th-1, Th-2, IRS, chemokine, and growth factor profiles, but especially the CIRS profile; b) an increase in a few selected cytokines/chemokines with systemic effects, including CCL5 and IL-1α, and IL-12p70 signaling; and c) an increased IRS/CIRS ratio.

IL-1α produced from peripheral blood may cross the BBB and get access to cortical brain cells [58]. The production of IL-1α is mediated by elevated levels of damage-associated molecular patterns, necrosis, and necroptotic stimuli during sterile inflammation [59, 60]. Moreover, MA exposure may enhance the production of HMGB1, a significant DAMP, that contributes to neuroinflammation [61] and may promote necrosis/necroptosis via dopaminergic, oxidative stress, and AGE-RAGE pathways [62, 63]. Additionally, peripheral and central IL-1α elevations play a significant role in CNS inflammation [60]. Interestingly, IL-1α may generate CCL5, another cytokine related with MAP [60]. As the levels of IL-1RA (which regulates IL-1-signaling; [64] are significantly reduced in MAP, the effects of IL-1α may become more prominent. CCL5 (RANTES) is a chemokine that may cross the BBB and reach the brain parenchyma, and CCL5 and its receptor are expressed in glia, whilst microglia and astrocytes are capable of producing CCL5 [65–68]. Elevated CCL5 expression in the central nervous system is associated with increased neuroinflammation, cortical synaptic excitability, and hyperalgesia, and is implicated in neuroinflammatory and neurodegenerative disease [65, 69, 70]. In addition, enhanced IL-12 signaling contributes to neuroinflammation and neurodegeneration [71–73], and IL-12 [73] is, as CCL27, implicated in inflammatory skin lesions.

Despite the fact that it is now well established that schizophrenia is an immunological condition and that first-episode schizophrenia and deficit schizophrenia are associated with IRS and CIRS activation [29, 74, 75], there are relatively few reports on the cytokine network in MAP. Yang et al. (2020) found that IL-6 and IL-8 were elevated in MAP patients and that sIL-2R was negatively correlated with positive symptoms [23]. Psychosis in amphetamine-dependent women during early cessation is associated with increases in IL-10, TNF-α, and IL-5 (the latter cytokine was not measurable in our research) [76]. MAP is also accompanied by neuroinflammatory aberrations in the AAC-thalamus circuits [77].

### Limitations

The study had several limitations that should be addressed. First, because the study is a case-control study, it is not possible to draw a firm conclusion about the causality of the associations. Second, self-reported drug use amounts are more difficult to interpret, and therefore we cannot conclude that MA may have “dose-dependent immunosuppressant effects.” Third, our MA dependence group included patients with high levels of dependence, reflecting a severe phenotype. Fourth, while we examined the effects of alcohol and cannabis use, no patients with other drug use were included, such as people who use opioids. Fifth, our results on the immune profiles in MA use, dependence, and MAP, deserve cross-validation in other countries and cultures. Sixth, the BioRad BioPlex kit is designed for use with plasma or serum, while we used whole blood. This is a deviation from the manufacturer’s recommended protocol. Nevertheless, in our hands, no major differences could be established between serum, plasma, and whole blood concentrations of cytokines. It should be noted that our observation that active MA use is accompanied by immunosuppression and increased levels of selected pro-inflammatory cytokines was made in peripheral blood. CNS samples can show more pro-inflammatory effects of MA during active use as well as in early abstinence.

Another question is whether immune markers associated with different stages of MA use (use, dependence, and MAP) could serve as diagnostic or prognostic indicators. Nevertheless, we would never recommend using cytokine/chemokines/growth factors as diagnostic biomarkers for MA use or MAP, because Elisa and Multiplex analyses have a large between-run analytical error especially at the low concentration levels as those detected in our study. Another clinical question is what types of interventions would be needed for people with MAP and those with MA dependency. One could of course argue that treatments which augment immune functions could counteract the immunosuppressive effects of MA. However, because immune activation may induce psychosis, it may not be the best strategy to activate the immune system. Future research should first examine if the MA-induced immunosuppression improves over time and additionally, how long it would take exactly before the immune system normalizes or improves.

### Conclusions

**Fig. 2:**
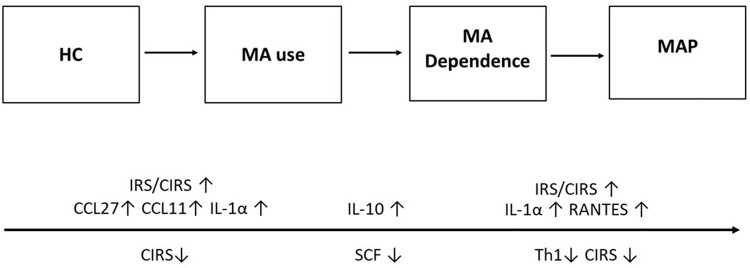
Summary of the findings of the current study. This figure shows the transitions from healthy controls to MA use, from MA use to MA dependence, and from the latter to MAP that are associated with changes in immune factors. Immune markers above the arrow are increased, under the arrow: decreased. HC healthy controls, MA methamphetamine, MAP MA-induced psychosis, IRS immune-inflammatory responses system, CIRS compensatory immunoregulatory system, IL interleukin, Th T helper, SCF stem cell factor.

Our results are summarized in Fig. 2. Long-term MA usage alters the immune system’s balance, leading to extensive immunosuppression (including increased IL-10 levels and decreased SCF levels) and the activation of certain cytokines, chemokines, and growth factors (including CCL27, IL-1α, and CCL11). Increases in IL-1α - CCL5 signaling, the IRS/CIRS ratio, and the severity of MA dependency all appear to work in concert to determine the onset of MAP. The results demonstrate that, despite the pervasive immunosuppression, elevated IL-1α - CCL5 signaling and maybe IL-12p70 signaling may be responsible for MAP.

### Supplementary information

Electronic Supplemenatary File

## Data Availability

The corresponding author (MM) will make the files used in the current study available upon receipt of an appropriate request.
