# Supplementary material for: Methamphetamine (MA) use and MA-induced psychosis are associated with increasing aberrations in the compensatory immunoregulatory system, interleukin-1α, and CCL5 levels
Source: Transl Psychiatry. 2023 Nov 23;13:361. doi: 10.1038/s41398-023-02645-6 (PMC10667231; doi:10.1038/s41398-023-02645-6)
Supplement: Supplementary file 1 — Electronic Supplemenatary File [file 41398_2023_2645_MOESM1_ESM.pdf]

Methamphetamine (MA) use and MA-induced psychosis are associated with increasing aberrations in the compensatory immunoregulatory system, interleukin-1α, and CCL5 levels

Running title: Methamphetamine impacts immunoregulation

(1-2) Rasmon Kalayasiri, M.D., (2) Kanokwan Dadwat, M.Sc., (2) Supaksorn Thika, M.Sc., (3) Sunee Sirivichayakul, Ph.D, (1,2,4-9) Michael Maes, M.D, Ph.D.

(1) Department of Psychiatry, Epidemiology of Psychiatric Disorders and Mental Health Research Unit, Faculty of Medicine, Chulalongkorn University, Bangkok, Thailand.

(2) King Chulalongkorn Memorial Hospital, Thai Red Cross Society, Bangkok, Thailand

(3) Division of Allergy and Clinical Immunology, Department of Medicine, Faculty of Medicine, Chulalongkorn University

(4) Cognitive Fitness and Technology Research Unit, Faculty of Medicine, Chulalongkorn University, Bangkok, Thailand

(5) Department of Psychiatry, Medical University of Plovdiv, Plovdiv, Bulgaria

(6) Research Institute, Medical University Plovdiv, Plovdiv, Bulgaria

(7) Kyung Hee University, 26 Kyungheedae-ro, Dongdaemun-gu, Seoul 02447, Korea

(8) Sichuan Provincial Center for Mental Health, Sichuan Provincial People’s Hospital, School of Medicine, University of Electronic Science and Technology of China, Chengdu 610072, China

(9) Key Laboratory of Psychosomatic Medicine, Chinese Academy of Medical Sciences, Chengdu, 610072, China

ESF, Table 1. Overview of the cytokines, chemokines and growth factors measured in the current study

Protein abbreviations	Gene Symbol	> OOR (%)	Protein name / alias
**IFN-α2**	**IFNA2**	42.0%	Interferon-α2
**IFN-γ**	**IFNG**	42.5%	Interferon-γ
**IL-1α**	**IL1A**	100%	Interleukin-1α
**IL-1β**	**IL1B**	99.4%	Interleukin-1β
**sIL-1RA**	**IL1RN**	100%	Soluble interleukin-1 receptor antagonist
**IL-2**	**IL2**	<7%	Interleukin-2
**IL-2RA**	**IL2RA**	100%	Soluble interleukin-2 receptor
**IL-3**	**IL3**	0%	Interleukin-3
**IL-4**	**IL4**	100%	Interleukin-4
**IL-5**	**IL5**	<7%	Interleukin-5
**IL-6**	**IL6**	8%	Interleukin-6

**IL-7**	**IL7**	10.9%	Interleukin-7
**IL-9**	**IL9**	100%	Interleukin-9
**IL-10**	**IL10**	42%	Interleukin-10
**IL-12p70**	**IL12RB1**	47%	Interleukin-12 p70
**IL-12p40**	**IL12RB1**	57.5%	Interleukin-12 p40
**IL-13**	**IL13**	88.5%	Interleukin-13
**IL-15**	**IL15**	<7%	Interleukin-15
**IL-16**	**IL16**	100%	Interleukin-16
**IL-17**	**IL17A**	8.6%	Interleukin-17
**IL-18**	**IL18**	100%	Interleukin-18
**TNF-α**	**TNF**	100%	Tumor necrosis factor-α
**TNF-β**	**LTA**	100%	Tumor necrosis factor-β or lymphotoxin-alpha (LT-α)
**TRAIL**	**TNFSF10**	100%	TNF-related apoptosis-inducing ligand (TRAIL) or tumor necrosis factor ligand superfamily member 10 (TNFSF10)
**LIF**	**LIF**	100%	Interleukin inhibitory factor
**MIF**	**MIF**	100%	Macrophage migration inhibitory factor-like protein (MIF) or glycosylation-inhibiting factor

**G-CSF**	**CSF3**	100%	Granulocyte colony stimulating factor (G-CSF) or colony stimulating factor 3 (CSF3)
**M-CSF**	**CSF1**	100%	Macrophage colony-stimulating factor (M-CSF) or colony stimulating factor 1 (CSF1)
**GM-CSF**	**CSF2**	<7%	Granulocyte-macrophage colony-stimulating factor (GM-CSF) or colony-stimulating factor 2 (CSF2)
**CCL2 or MCP1**	**CCL2**	100%	C-C motif chemokine ligand 2 (CCL2) or monocyte chemoattractant protein 1 (MCP1)
**CCL3 or MIP-1α**	**CCL3**	99.4%	C-C motif Chemokine ligand 3 (CCL3) or macrophage inflammatory protein 1-alpha (MIP-1α)
**CCL4 or MIP-1β**	**CCL4**	100%	C-C motif chemokine ligand 4 (CCL4) or macrophage inflammatory protein 1β (MIP-1β) or lymphocyte activation gene 1 protein
**CCL5 or RANTES**	**CCL5**	71.8%	C-C motif chemokine ligand 5 (CCL5) or regulated upon activation, normally T-expressed, and presumably Secreted (RANTES)
**CCL7 or MCP3**	**CCL7**	78.2%	C-C motif chemokine ligand 7 (CCL7) or monocyte-chemotactic protein 3 (MCP3).
**CCL11 or Eotaxin**	**CCL11**	100%	C-C motif chemokine ligand 11 (CCL11) or eosinophil chemotactic protein
**CCL27 or CTACK**	**CCL27**	100%	C-C motif chemokine ligand 27 (CCL27) or cutaneous T-cell attracting chemokine (CTACK)
**CXCL1 or GRO-α**	**CXCL1**	83.9%	C-X-C motif chemokine 1 (CXCL1) or growth-regulated alpha protein (GRO)
**CXCL8 or IL-8**	**CXCL8**	99.4%	C-X-C motif chemokine ligand 8 (CXCL8) or interleukin-8 (IL-8)
**CXCL9 or MIG**	**CXCL9**	100%	C-X-C motif chemokine ligand 9 (CXCL9) or monokine induced by gamma interferon (MIG)
**CXCL10 or IP10**	**CXCL10**	100%	C-X-C motif chemokine ligand 10 (CXCL10) or Interferon gamma-induced protein 10 (IP10)
**CXCL12 or SDF-1α**	**CXCL12**	100%	C-X-C motif chemokine 12 (CXCL12) or stromal cell-derived factor 1 (SDF-1α)

**FGF**	**FGF2**	100%	Fibroblast growth factor 2 (FGF) or basic fibroblast growth factor
**HGF or SF**	**HGF**	100%	Hepatocyte growth factor (HGF) or scatter factor (SF)
**NGF**	**NGF**	<7%	β-nerve growth factor (NGF)
**PDGF**	**PDGFA**	99.4%	Platelet derived growth factor (PDGF)
**SCF**	**KITLG**	100%	Stem cell factor (SCF) or Kit ligand (KITLG)
**SCGF-β or CLEC11A**	**CLEC11A**	100%	Stem cell growth factor (SCGF) or C-type lectin domain family 11 member A (CLEC11A)
**VEGF**	**VEGFA**	15.5%	Vascular endothelial growth factor (VEGF)

ESF, Table 2. Composite scores used in the present study

Immune Profiles	Members
**M1 macrophage**	IL-1α, IL-1β, sIL-1RA, IL-6, TNF-α, CXCL8, CXCL10, CCL2, CCL3, CCL4
**T helper-1**	IL-2, sIL-2R, IFN-α, IFN-γ, IL-12p70, IL12p40, IL-16
**T helper-2**	IL-4, IL-9, IL-13
**IRS**	IL-1α, IL-1β, IL-6, LIF, IL-7, TNF-α, TNF-β, TRAIL, IFN-α2, IFN-γ, IL-12p70, IL-16, IL-18, G-CSF, M-CSF, CCL2, CCL3, CCL4, CCL5, CCL7, CC11, CCL, 27, CXCL1, CXCL8, CXCL9, CXCL10, CXCL12
**CIRS**	IL-4, IL-9, IL-10, sIL-1RA, IL-12p40, sIL-2R
**Chemokines**	All CCL and all CXCL members
**Growth factors**	FGF, HGF, PDGF, SCF, SCGF, VEGF

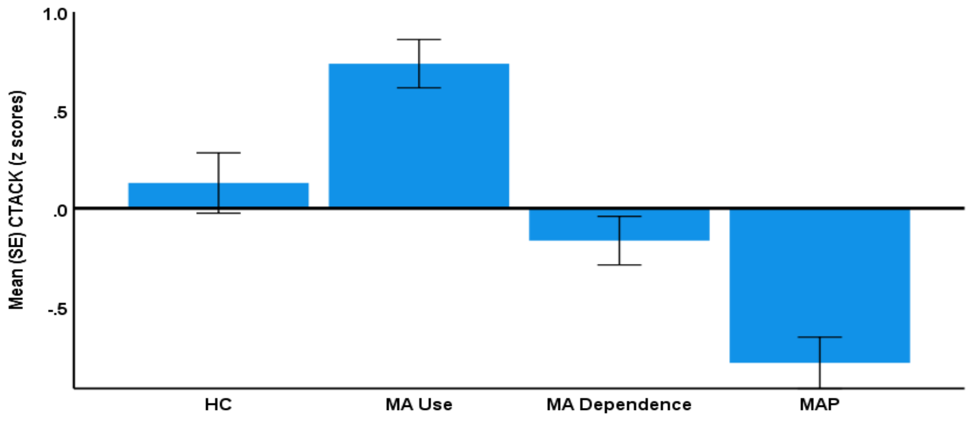

Figure S1. CCL27 or CTACK levels in healthy controls (HC) and people with methamphetamine (MA) use, MA dependence, and MA-induced psychosis (MAP) (F=23.87, df=3/164, p<0.001).

Pairwise Comparisons

Dependent Variable: CTACK

(I) HC_Abuse_ Dependence_ Psychosis	(J) HC_Abuse_ Dependence_ Psychosis	Mean Difference (I-J)	Std. Error	Sig.[b]	95% Confidence Interval for Difference[b]	
					Lower Bound	Upper Bound
0	1	-.608*	0.202	0.003	-1.006	-0.209
	2	0.293	0.200	0.145	-0.102	0.688
	3	.915*	0.199	<.001	0.523	1.308
1	0	.608*	0.202	0.003	0.209	1.006
	2	.901*	0.174	<.001	0.557	1.245
	3	1.523*	0.185	<.001	1.158	1.888
2	0	-0.293	0.200	0.145	-0.688	0.102
	1	-.901*	0.174	<.001	-1.245	-0.557
	3	.622*	0.181	<.001	0.265	0.980
3	0	-.915*	0.199	<.001	-1.308	-0.523
	1	-1.523*	0.185	<.001	-1.888	-1.158
	2	-.622*	0.181	<.001	-0.980	-0.265

Based on estimated marginal means

*. The mean difference is significant at the 0.05 level.

b. Adjustment for multiple comparisons: Least Significant Difference (equivalent to no adjustments).

HC=0, MA use=1, MA dependence=2, MAP=3

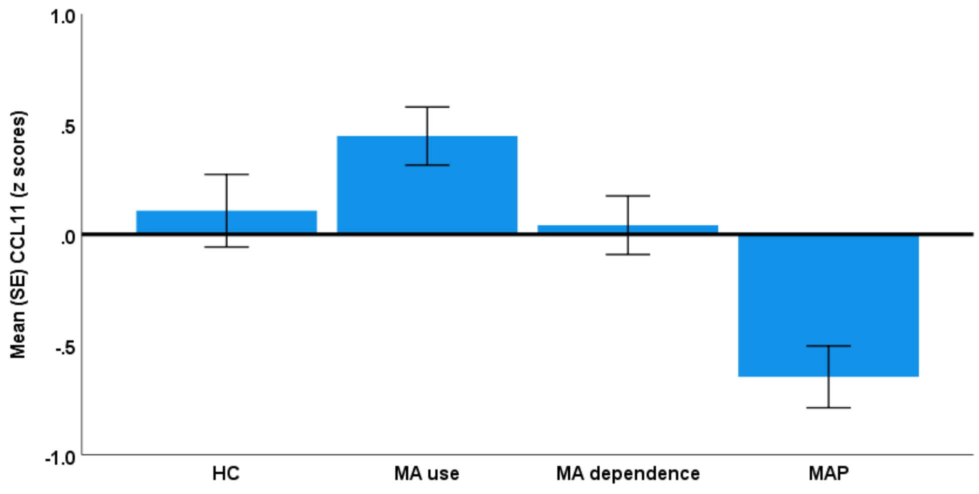

Figure S2. CCL11 levels in healthy controls (HC) and people with methamphetamine (MA) use, MA dependence, and MA-induced psychosis (MAP) (F=10.64, df=3/164, p<0.001).

Pairwise Comparisons

Dependent Variable: CCL11

(I) HC_Abuse_ Dependence_ Psychosis	(J) HC_Abuse_ Dependence_ Psychosis	Mean Difference (I-J)	Std. Error	Sig.[b]	95% Confidence Interval for Difference[b]	
					Lower Bound	Upper Bound
0	1	-0.339	0.217	0.120	-0.767	0.089
	2	0.066	0.215	0.760	-0.358	0.489
	3	.754*	0.213	0.001	0.333	1.175
1	0	0.339	0.217	0.120	-0.089	0.767
	2	.404*	0.187	0.032	0.036	0.773
	3	1.092*	0.198	0.000	0.701	1.484
2	0	-0.066	0.215	0.760	-0.489	0.358
	1	-.404*	0.187	0.032	-0.773	-0.036
	3	.688*	0.194	0.001	0.305	1.071
3	0	-.754*	0.213	0.001	-1.175	-0.333
	1	-1.092*	0.198	0.000	-1.484	-0.701
	2	-.688*	0.194	0.001	-1.071	-0.305

Based on estimated marginal means

*. The mean difference is significant at the 0.05 level.

b. Adjustment for multiple comparisons: Least Significant Difference (equivalent to no adjustments).

HC=0, MA use=1, MA dependence=2, MAP=3

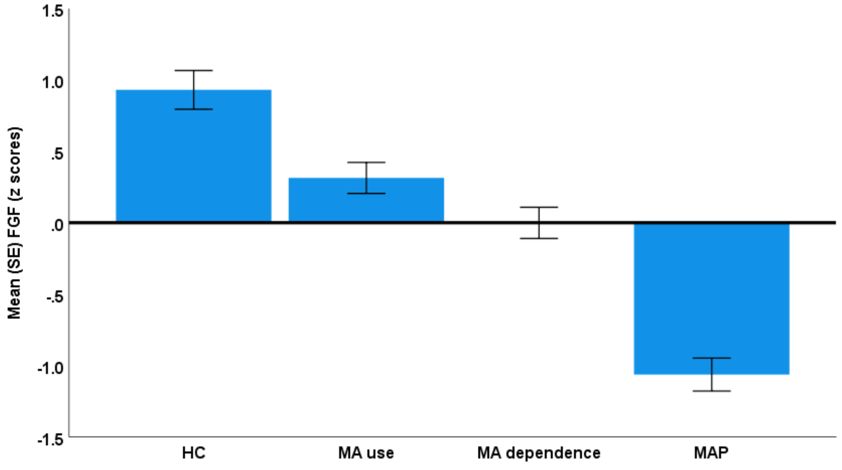

Figure S3. FGF levels in healthy controls (HC) and people with methamphetamine (MA) use, MA dependence, and MA-induced psychosis (MAP) ($F=47.71$, $df=3/164$, $p<0.001$).

Pairwise Comparisons

Dependent Variable: FGF

(I) HC_Abuse_ Dependence_ Psychosis	(J) HC_Abuse_ Dependence_ Psychosis	Mean Difference (I-J)	Std. Error	Sig.[b]	95% Confidence Interval for Difference[b]	
					Lower Bound	Upper Bound
0	1	.617*	0.178	0.001	0.264	0.969
	2	.932*	0.177	0.000	0.583	1.280
	3	1.994*	0.176	0.000	1.647	2.341
1	0	-.617*	0.178	0.001	-0.969	-0.264
	2	.315*	0.154	0.042	0.011	0.619
	3	1.377*	0.163	0.000	1.055	1.699
2	0	-.932*	0.177	0.000	-1.280	-0.583
	1	-.315*	0.154	0.042	-0.619	-0.011
	3	1.062*	0.160	0.000	0.747	1.378
3	0	-1.994*	0.176	0.000	-2.341	-1.647
	1	-1.377*	0.163	0.000	-1.699	-1.055
	2	-1.062*	0.160	0.000	-1.378	-0.747

Based on estimated marginal means

*. The mean difference is significant at the 0.05 level.

b. Adjustment for multiple comparisons: Least Significant Difference (equivalent to no adjustments).

HC=0, MA use=1, MA dependence=2, MAP=3

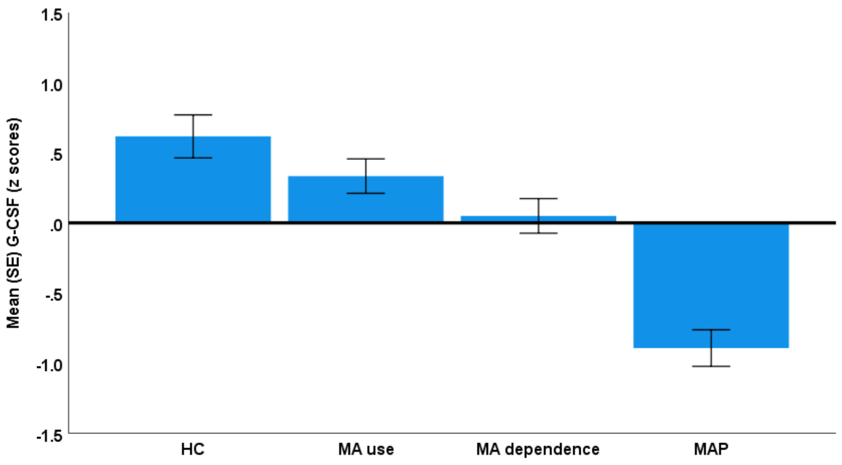

Figure S4. G-CSF levels in healthy controls (HC) and people with methamphetamine (MA) use, MA dependence, and MA-induced psychosis (MAP) (F=23.77, df=3/164, p<0.001).

Pairwise Comparisons

Dependent Variable: G-CSF

(I) HC_Abuse_ Dependence_ Psychosis	(J) HC_Abuse_ Dependence_ Psychosis	Mean Difference (I-J)	Std. Error	Sig.[b]	95% Confidence Interval for Difference[b]	
					Lower Bound	Upper Bound
0	1	0.283	0.202	0.162	-0.115	0.681
	2	.567*	0.200	0.005	0.173	0.961
	3	1.509*	0.198	0.000	1.118	1.901
1	0	-0.283	0.202	0.162	-0.681	0.115
	2	0.284	0.174	0.105	-0.060	0.627
	3	1.226*	0.184	0.000	0.862	1.590
2	0	-.567*	0.200	0.005	-0.961	-0.173
	1	-0.284	0.174	0.105	-0.627	0.060
	3	.942*	0.181	0.000	0.586	1.299
3	0	-1.509*	0.198	0.000	-1.901	-1.118
	1	-1.226*	0.184	0.000	-1.590	-0.862
	2	-.942*	0.181	0.000	-1.299	-0.586

Based on estimated marginal means

*. The mean difference is significant at the 0.05 level.

b. Adjustment for multiple comparisons: Least Significant Difference (equivalent to no adjustments).

HC=0, MA use=1, MA dependence=2, MAP=3

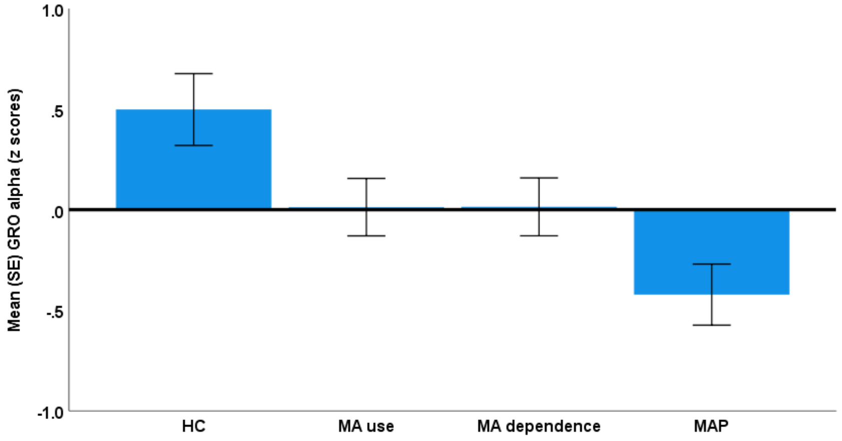

Figure S5. CXCL1 (GRO-α) levels in healthy controls (HC) and people with methamphetamine (MA) use, MA dependence, and MA-induced psychosis (MAP) ($F=5.33$, $df=3/164$, $p=0.002$).

Pairwise Comparisons

Dependent Variable: GRO-α

(I) HC_Abuse_ Dependence_ Psychosis	(J) HC_Abuse_ Dependence_ Psychosis	Mean Difference (I-J)	Std. Error	Sig.[b]	95% Confidence Interval for Difference[b]	
					Lower Bound	Upper Bound
0	1	.485*	0.235	0.040	0.022	0.949
	2	.484*	0.233	0.039	0.025	0.943
	3	.920*	0.231	0.000	0.464	1.377
1	0	-.485*	0.235	0.040	-0.949	-0.022
	2	-0.002	0.202	0.994	-0.401	0.398
	3	.435*	0.215	0.044	0.011	0.859
2	0	-.484*	0.233	0.039	-0.943	-0.025
	1	0.002	0.202	0.994	-0.398	0.401
	3	.437*	0.210	0.040	0.021	0.852
3	0	-.920*	0.231	0.000	-1.377	-0.464
	1	-.435*	0.215	0.044	-0.859	-0.011
	2	-.437*	0.210	0.040	-0.852	-0.021

Based on estimated marginal means

*. The mean difference is significant at the 0.05 level.

b. Adjustment for multiple comparisons: Least Significant Difference (equivalent to no adjustments).

HC=0, MA use=1, MA dependence=2, MAP=3

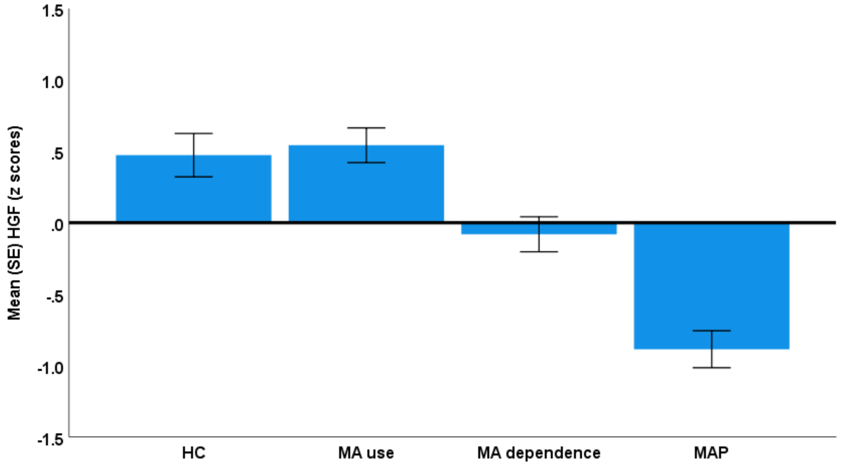

Figure S6. HGF levels in healthy controls (HC) and people with methamphetamine (MA) use, MA dependence, and MA-induced psychosis (MAP) (F=25.43, df=3/164, p<0.001).

Pairwise Comparisons

Dependent Variable: HGF

(I) HC_Abuse_ Dependence_ Psychosis	(J) HC_Abuse_ Dependence_ Psychosis	Mean Difference (I-J)	Std. Error	Sig.[b]	95% Confidence Interval for Difference[b]	
					Lower Bound	Upper Bound
0	1	-0.070	0.200	0.728	-0.464	0.325
	2	.554*	0.198	0.006	0.163	0.945
	3	1.359*	0.197	0.000	0.971	1.748
1	0	0.070	0.200	0.728	-0.325	0.464
	2	.624*	0.172	0.000	0.283	0.964
	3	1.429*	0.183	0.000	1.068	1.790
2	0	-.554*	0.198	0.006	-0.945	-0.163
	1	-.624*	0.172	0.000	-0.964	-0.283
	3	.805*	0.179	0.000	0.452	1.159
3	0	-1.359*	0.197	0.000	-1.748	-0.971
	1	-1.429*	0.183	0.000	-1.790	-1.068
	2	-.805*	0.179	0.000	-1.159	-0.452

Based on estimated marginal means

*. The mean difference is significant at the 0.05 level.

b. Adjustment for multiple comparisons: Least Significant Difference (equivalent to no adjustments).

HC=0, MA use=1, MA dependence=2, MAP=3

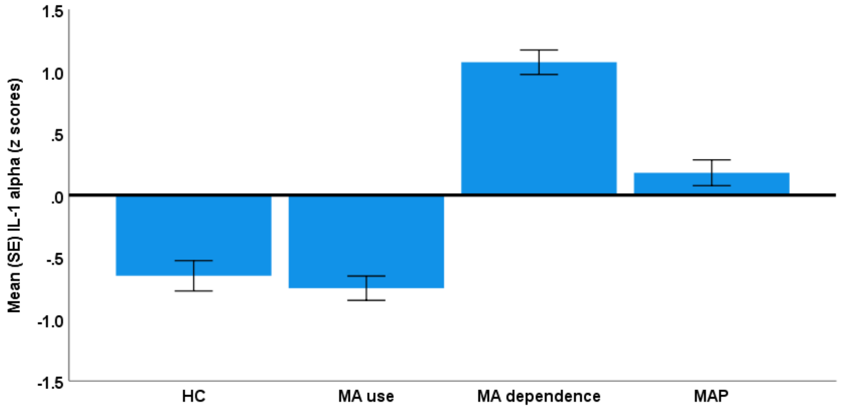

Figure S7. IL-1α levels in healthy controls (HC) and people with methamphetamine (MA) use, MA dependence, and MA-induced psychosis (MAP) (F=69.76, df=3/164, p<0.001).

Pairwise Comparisons

Dependent Variable: IL-1α

(I) HC_Abuse_Dependence_Psychosis	(J) HC_Abuse_Dependence_Psychosis	Mean Difference (I-J)	Std. Error	Sig.[b]	95% Confidence Interval for Difference[b] Lower Bound	Upper Bound
0	1	0.100	0.161	0.534	-0.218	0.419
	2	-1.725*	0.160	0.000	-2.040	-1.410
	3	-.832*	0.159	0.000	-1.145	-0.519
1	0	-0.100	0.161	0.534	-0.419	0.218
	2	-1.825*	0.139	0.000	-2.100	-1.551
	3	-.933*	0.147	0.000	-1.224	-0.642
2	0	1.725*	0.160	0.000	1.410	2.040
	1	1.825*	0.139	0.000	1.551	2.100
	3	.892*	0.144	0.000	0.607	1.178
3	0	.832*	0.159	0.000	0.519	1.145
	1	.933*	0.147	0.000	0.642	1.224
	2	-.892*	0.144	0.000	-1.178	-0.607

Based on estimated marginal means

*. The mean difference is significant at the 0.05 level.

b. Adjustment for multiple comparisons: Least Significant Difference (equivalent to no adjustments).

HC=0, MA use=1, MA dependence=2, MAP=3

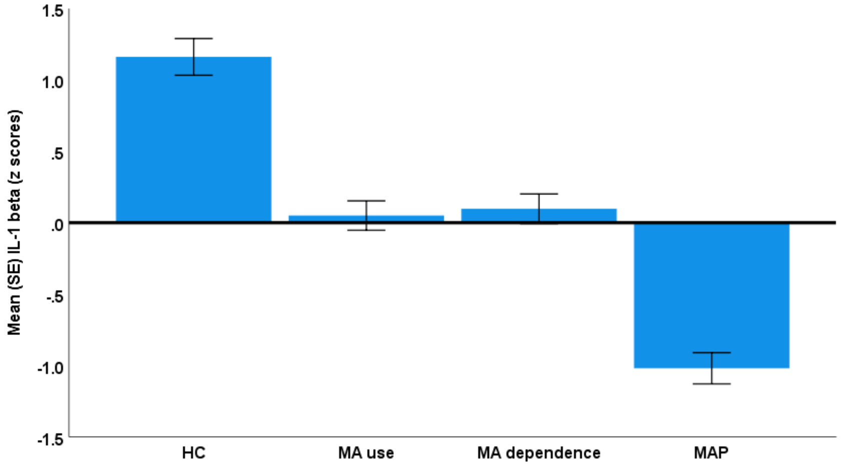

Figure S8. IL-1β levels in healthy controls (HC) and people with methamphetamine (MA) use, MA dependence, and MA-induced psychosis (MAP) ($F=58.11$, $df=3/164$, $p<0.001$).

Pairwise Comparisons

Dependent Variable: IL-1β

(I) HC_Abuse_ Dependence_ Psychosis	(J) HC_Abuse_ Dependence_ Psychosis	Mean Difference (I-J)	Std. Error	Sig.[b]	95% Confidence Interval for Difference[b]	
					Lower Bound	Upper Bound
0	1	1.111*	0.169	0.000	0.777	1.446
	2	1.064*	0.168	0.000	0.733	1.395
	3	2.181*	0.167	0.000	1.852	2.510
1	0	-1.111*	0.169	0.000	-1.446	-0.777
	2	-0.047	0.146	0.746	-0.336	0.241
	3	1.070*	0.155	0.000	0.764	1.375
2	0	-1.064*	0.168	0.000	-1.395	-0.733
	1	0.047	0.146	0.746	-0.241	0.336
	3	1.117*	0.152	0.000	0.817	1.416
3	0	-2.181*	0.167	0.000	-2.510	-1.852
	1	-1.070*	0.155	0.000	-1.375	-0.764
	2	-1.117*	0.152	0.000	-1.416	-0.817

Based on estimated marginal means

*. The mean difference is significant at the 0.05 level.

b. Adjustment for multiple comparisons: Least Significant Difference (equivalent to no adjustments).

HC=0, MA use=1, MA dependence=2, MAP=3

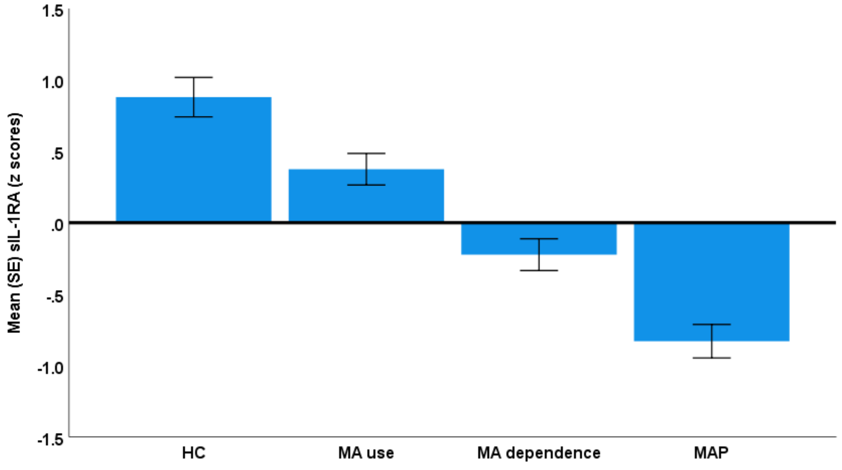

Figure S9. sIL-1RA levels in healthy controls (HC) and people with methamphetamine (MA) use, MA dependence, and MA-induced psychosis (MAP) (F=35.87, df=3/164, p<0.001).

Pairwise Comparisons

Dependent Variable: sIL-1RA

(I) HC_Abuse_ Dependence_ Psychosis	(J) HC_Abuse_ Dependence_ Psychosis	Mean Difference (I-J)	Std. Error	Sig.[b]	95% Confidence Interval for Difference[b]	
					Lower Bound	Upper Bound
0	1	.505*	0.182	0.006	0.146	0.864
	2	1.103*	0.180	0.000	0.748	1.459
	3	1.709*	0.179	0.000	1.356	2.062
1	0	-.505*	0.182	0.006	-0.864	-0.146
	2	.598*	0.157	0.000	0.289	0.908
	3	1.204*	0.166	0.000	0.876	1.533
2	0	-1.103*	0.180	0.000	-1.459	-0.748
	1	-.598*	0.157	0.000	-0.908	-0.289
	3	.606*	0.163	0.000	0.284	0.927
3	0	-1.709*	0.179	0.000	-2.062	-1.356
	1	-1.204*	0.166	0.000	-1.533	-0.876
	2	-.606*	0.163	0.000	-0.927	-0.284

Based on estimated marginal means

*. The mean difference is significant at the 0.05 level.

b. Adjustment for multiple comparisons: Least Significant Difference (equivalent to no adjustments).

HC=0, MA use=1, MA dependence=2, MAP=3

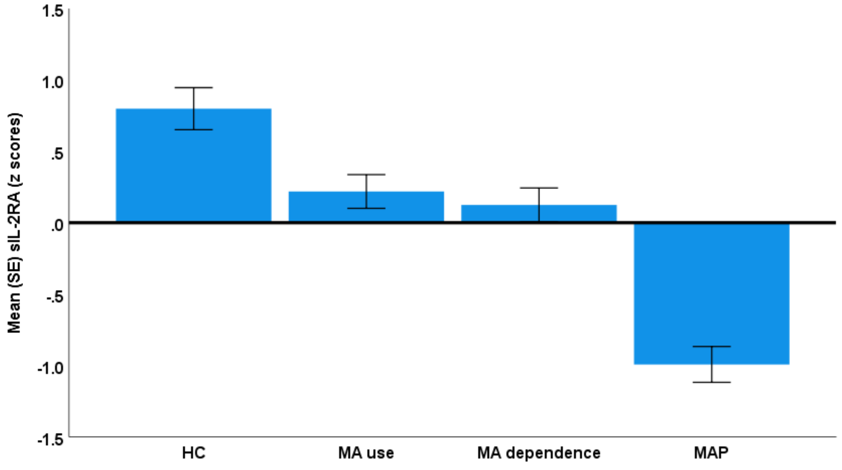

Figure S10. sIL-2RA levels in healthy controls (HC) and people with methamphetamine (MA) use, MA dependence, and MA-induced psychosis (MAP) (F=32.87, df=3/164, p<0.001).

Pairwise Comparisons

Dependent Variable: sIL-2RA

(I) HC_Abuse_ Dependence_ Psychosis	(J) HC_Abuse_ Dependence_ Psychosis	Mean Difference (I-J)	Std. Error	Sig.[b]	95% Confidence Interval for Difference[b]	
					Lower Bound	Upper Bound
0	1	.580*	0.194	0.003	0.197	0.963
	2	.674*	0.192	0.001	0.295	1.053
	3	1.792*	0.191	0.000	1.415	2.168
1	0	-.580*	0.194	0.003	-0.963	-0.197
	2	0.094	0.167	0.575	-0.236	0.424
	3	1.211*	0.177	0.000	0.861	1.562
2	0	-.674*	0.192	0.001	-1.053	-0.295
	1	-0.094	0.167	0.575	-0.424	0.236
	3	1.117*	0.174	0.000	0.774	1.460
3	0	-1.792*	0.191	0.000	-2.168	-1.415
	1	-1.211*	0.177	0.000	-1.562	-0.861
	2	-1.117*	0.174	0.000	-1.460	-0.774

Based on estimated marginal means

*. The mean difference is significant at the 0.05 level.

b. Adjustment for multiple comparisons: Least Significant Difference (equivalent to no adjustments).

HC=0, MA use=1, MA dependence=2, MAP=3

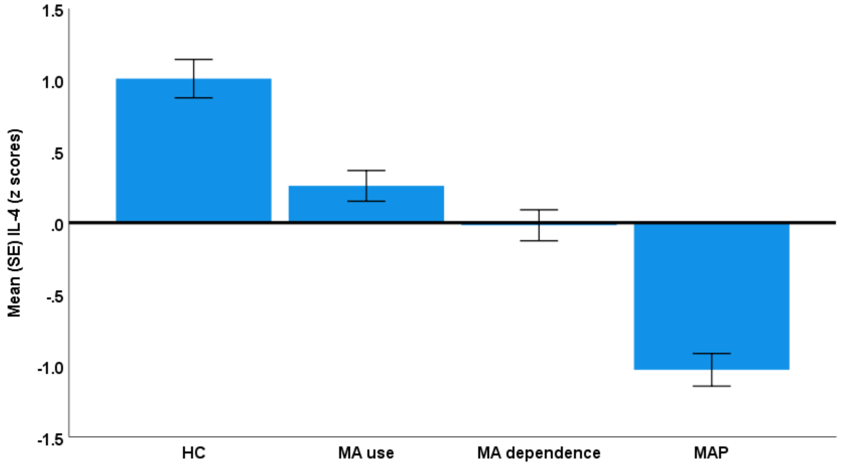

Figure S11. IL-4 levels in healthy controls (HC) and people with methamphetamine (MA) use, MA dependence, and MA-induced psychosis (MAP) ($F=48.96$, $df=3/164$, $p<0.001$).

Pairwise Comparisons

Dependent Variable: IL-4

(I) HC_Abuse_ Dependence_ Psychosis	(J) HC_Abuse_ Dependence_ Psychosis	Mean Difference (I-J)	Std. Error	Sig.[b]	95% Confidence Interval for Difference[b]	
					Lower Bound	Upper Bound
0	1	.751*	0.177	0.000	0.402	1.100
	2	1.027*	0.175	0.000	0.682	1.373
	3	2.040*	0.174	0.000	1.696	2.383
1	0	-.751*	0.177	0.000	-1.100	-0.402
	2	0.276	0.152	0.072	-0.025	0.577
	3	1.289*	0.162	0.000	0.969	1.608
2	0	-1.027*	0.175	0.000	-1.373	-0.682
	1	-0.276	0.152	0.072	-0.577	0.025
	3	1.013*	0.158	0.000	0.700	1.325
3	0	-2.040*	0.174	0.000	-2.383	-1.696
	1	-1.289*	0.162	0.000	-1.608	-0.969
	2	-1.013*	0.158	0.000	-1.325	-0.700

Based on estimated marginal means

*. The mean difference is significant at the 0.05 level.

b. Adjustment for multiple comparisons: Least Significant Difference (equivalent to no adjustments).

HC=0, MA use=1, MA dependence=2, MAP=3

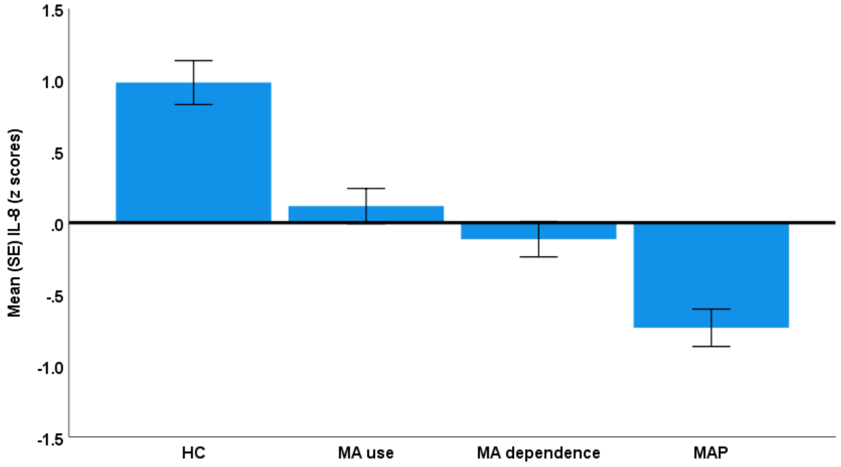

Figure S12.CXCL8 or IL-8 levels in healthy controls (HC) and people with methamphetamine (MA) use, MA dependence, and MA-induced psychosis (MAP) (F=25.29, df=3/164, p<0.001).

Pairwise Comparisons

Dependent Variable: IL-8

(I) HC_Abuse_ Dependence_ Psychosis	(J) HC_Abuse_ Dependence_ Psychosis	Mean Difference (I-J)	Std. Error	Sig.[b]	95% Confidence Interval for Difference[b]	
					Lower Bound	Upper Bound
0	1	.865*	0.203	0.000	0.465	1.265
	2	1.097*	0.201	0.000	0.701	1.493
	3	1.717*	0.199	0.000	1.324	2.111
1	0	-.865*	0.203	0.000	-1.265	-0.465
	2	0.232	0.175	0.186	-0.113	0.577
	3	.852*	0.185	0.000	0.487	1.218
2	0	-1.097*	0.201	0.000	-1.493	-0.701
	1	-0.232	0.175	0.186	-0.577	0.113
	3	.621*	0.181	0.001	0.262	0.979
3	0	-1.717*	0.199	0.000	-2.111	-1.324
	1	-.852*	0.185	0.000	-1.218	-0.487
	2	-.621*	0.181	0.001	-0.979	-0.262

Based on estimated marginal means

*. The mean difference is significant at the 0.05 level.

b. Adjustment for multiple comparisons: Least Significant Difference (equivalent to no adjustments).

HC=0, MA use=1, MA dependence=2, MAP=3

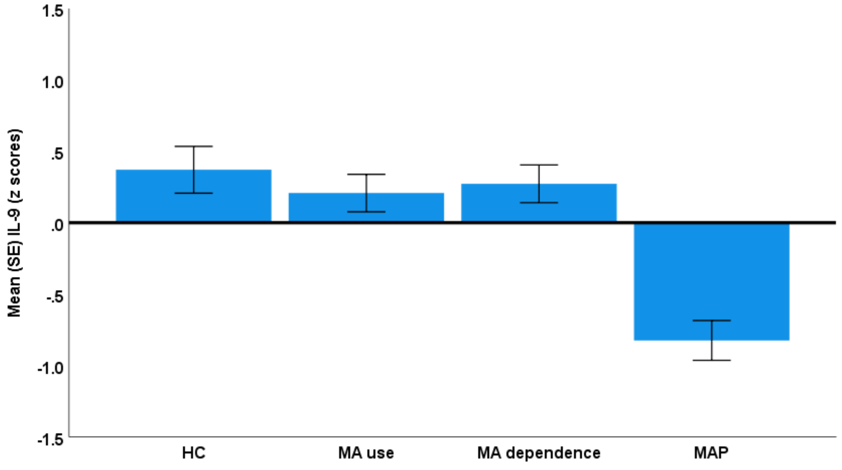

Figure S13. IL-9 levels in healthy controls (HC) and people with methamphetamine (MA) use, MA dependence, and MA-induced psychosis (MAP) (F=15.67, df=3/164, p<0.001).

Pairwise Comparisons

Dependent Variable: IL-9

(I) HC_Abuse_ Dependence_ Psychosis	(J) HC_Abuse_ Dependence_ Psychosis	Mean Difference (I-J)	Std. Error	Sig.[b]	95% Confidence Interval for Difference[b]	
					Lower Bound	Upper Bound
0	1	0.163	0.216	0.451	-0.263	0.589
	2	0.098	0.214	0.646	-0.323	0.520
	3	1.195*	0.212	0.000	0.776	1.614
1	0	-0.163	0.216	0.451	-0.589	0.263
	2	-0.065	0.186	0.728	-0.432	0.302
	3	1.032*	0.197	0.000	0.643	1.422
2	0	-0.098	0.214	0.646	-0.520	0.323
	1	0.065	0.186	0.728	-0.302	0.432
	3	1.097*	0.193	0.000	0.716	1.478
3	0	-1.195*	0.212	0.000	-1.614	-0.776
	1	-1.032*	0.197	0.000	-1.422	-0.643
	2	-1.097*	0.193	0.000	-1.478	-0.716

Based on estimated marginal means

*. The mean difference is significant at the 0.05 level.

b. Adjustment for multiple comparisons: Least Significant Difference (equivalent to no adjustments).

HC=0, MA use=1, MA dependence=2, MAP=3

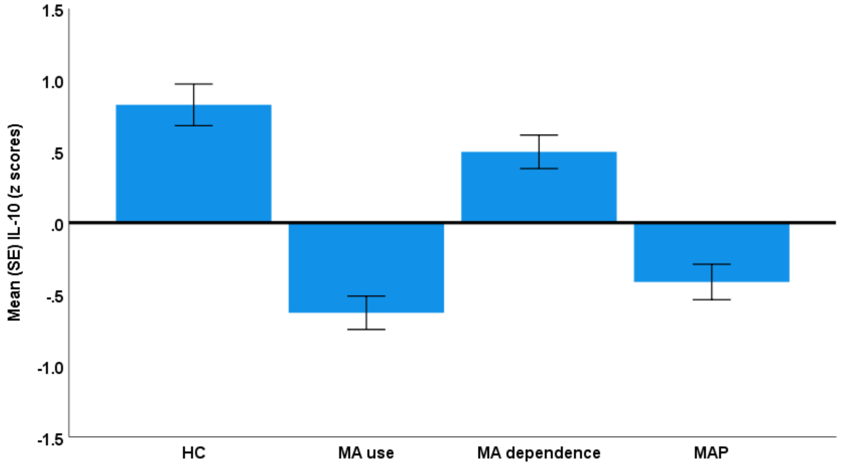

Figure S14. IL-10 levels in healthy controls (HC) and people with methamphetamine (MA) use, MA dependence, and MA-induced psychosis (MAP) (F=30.46, df=3/164, p<0.001).

Pairwise Comparisons

Dependent Variable: IL-10

(I) HC_Abuse_ Dependence_ Psychosis	(J) HC_Abuse_ Dependence_ Psychosis	Mean Difference (I-J)	Std. Error	Sig.[b]	95% Confidence Interval for Difference[b]	
					Lower Bound	Upper Bound
0	1	1.456*	0.192	0.000	1.077	1.835
	2	0.329	0.190	0.086	-0.047	0.704
	3	1.240*	0.189	0.000	0.867	1.614
1	0	-1.456*	0.192	0.000	-1.835	-1.077
	2	-1.127*	0.166	0.000	-1.454	-0.800
	3	-0.216	0.176	0.221	-0.562	0.131
2	0	-0.329	0.190	0.086	-0.704	0.047
	1	1.127*	0.166	0.000	0.800	1.454
	3	.912*	0.172	0.000	0.572	1.251
3	0	-1.240*	0.189	0.000	-1.614	-0.867
	1	0.216	0.176	0.221	-0.131	0.562
	2	-.912*	0.172	0.000	-1.251	-0.572

Based on estimated marginal means

*. The mean difference is significant at the 0.05 level.

b. Adjustment for multiple comparisons: Least Significant Difference (equivalent to no adjustments).

HC=0, MA use=1, MA dependence=2, MAP=3

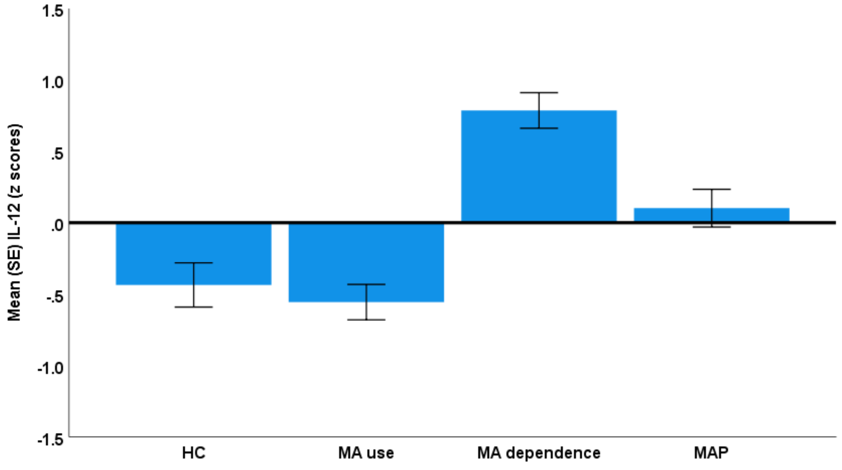

Figure S15. IL-12 p70 levels in healthy controls (HC) and people with methamphetamine (MA) use, MA dependence, and MA-induced psychosis (MAP) (F=22.87, df=3/164, p<0.001).

Pairwise Comparisons

Dependent Variable: IL-12p70

(I) HC_Abuse_ Dependence_ Psychosis	(J) HC_Abuse_ Dependence_ Psychosis	Mean Difference (I-J)	Std. Error	Sig.[b]	95% Confidence Interval for Difference[b]	
					Lower Bound	Upper Bound
0	1	0.120	0.204	0.556	-0.282	0.523
	2	-1.222*	0.202	0.000	-1.621	-0.823
	3	-.538*	0.201	0.008	-0.934	-0.142
1	0	-0.120	0.204	0.556	-0.523	0.282
	2	-1.343*	0.176	0.000	-1.690	-0.995
	3	-.658*	0.186	0.001	-1.026	-0.290
2	0	1.222*	0.202	0.000	0.823	1.621
	1	1.343*	0.176	0.000	0.995	1.690
	3	.684*	0.183	0.000	0.324	1.045
3	0	.538*	0.201	0.008	0.142	0.934
	1	.658*	0.186	0.001	0.290	1.026
	2	-.684*	0.183	0.000	-1.045	-0.324

Based on estimated marginal means

*. The mean difference is significant at the 0.05 level.

b. Adjustment for multiple comparisons: Least Significant Difference (equivalent to no adjustments).

HC=0, MA use=1, MA dependence=2, MAP=3

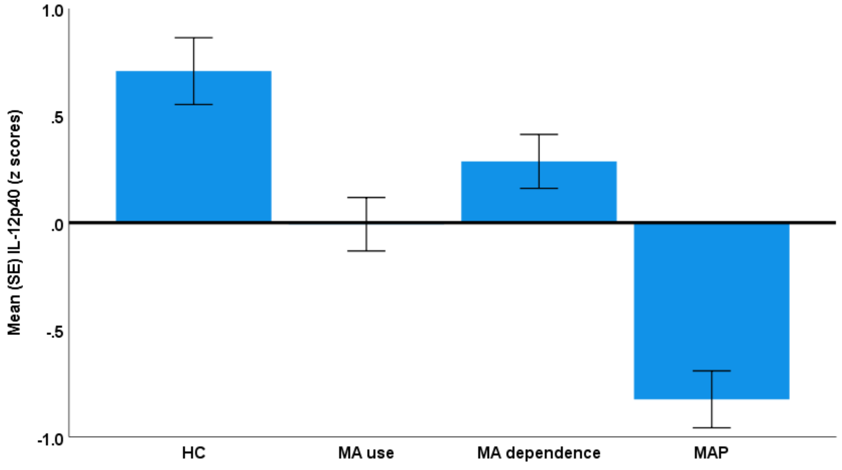

Figure S16. IL-12p40 levels in healthy controls (HC) and people with methamphetamine (MA) use, MA dependence, and MA-induced psychosis (MAP) (F=22.09, df=3/164, p<0.001).

Pairwise Comparisons

Dependent Variable: IL-12p40

(I) HC_Abuse_ Dependence_ Psychosis	(J) HC_Abuse_ Dependence_ Psychosis	Mean Difference (I-J)	Std. Error	Sig.[b]	95% Confidence Interval for Difference[b]	
					Lower Bound	Upper Bound
0	1	.715*	0.205	0.001	0.309	1.120
	2	.421*	0.203	0.040	0.020	0.823
	3	1.533*	0.202	0.000	1.133	1.932
1	0	-.715*	0.205	0.001	-1.120	-0.309
	2	-0.293	0.177	0.100	-0.643	0.056
	3	.818*	0.188	0.000	0.447	1.189
2	0	-.421*	0.203	0.040	-0.823	-0.020
	1	0.293	0.177	0.100	-0.056	0.643
	3	1.111*	0.184	0.000	0.748	1.475
3	0	-1.533*	0.202	0.000	-1.932	-1.133
	1	-.818*	0.188	0.000	-1.189	-0.447
	2	-1.111*	0.184	0.000	-1.475	-0.748

Based on estimated marginal means

*. The mean difference is significant at the 0.05 level.

b. Adjustment for multiple comparisons: Least Significant Difference (equivalent to no adjustments).

HC=0, MA use=1, MA dependence=2, MAP=3

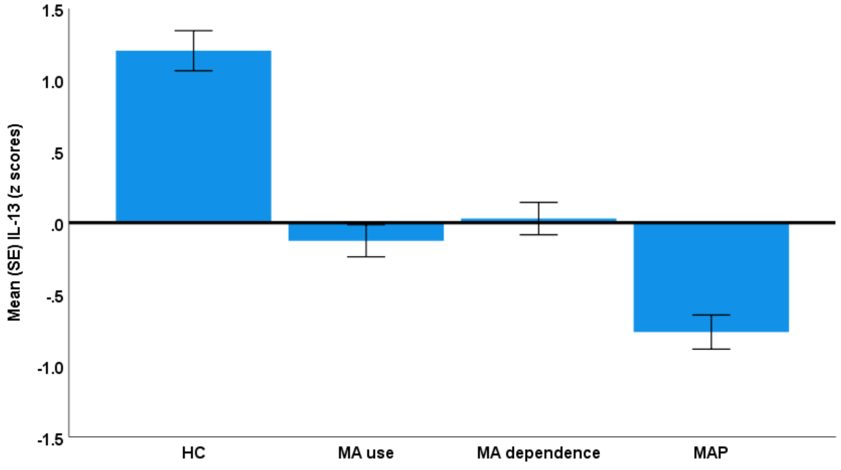

Figure S17. IL-13 levels in healthy controls (HC) and people with methamphetamine (MA) use, MA dependence, and MA-induced psychosis (MAP) (F=39.64, df=3/164, p<0.001).

Pairwise Comparisons

Dependent Variable: IL-13

(I) HC_Abuse_ Dependence_ Psychosis	(J) HC_Abuse_ Dependence_ Psychosis	Mean Difference (I-J)	Std. Error	Sig.[b]	95% Confidence Interval for Difference[b]	
					Lower Bound	Upper Bound
0	1	1.331*	0.185	0.000	0.966	1.696
	2	1.175*	0.183	0.000	0.814	1.537
	3	1.970*	0.182	0.000	1.611	2.330
1	0	-1.331*	0.185	0.000	-1.696	-0.966
	2	-0.156	0.159	0.331	-0.470	0.159
	3	.640*	0.169	0.000	0.306	0.973
2	0	-1.175*	0.183	0.000	-1.537	-0.814
	1	0.156	0.159	0.331	-0.159	0.470
	3	.795*	0.166	0.000	0.468	1.122
3	0	-1.970*	0.182	0.000	-2.330	-1.611
	1	-.640*	0.169	0.000	-0.973	-0.306
	2	-.795*	0.166	0.000	-1.122	-0.468

Based on estimated marginal means

*. The mean difference is significant at the 0.05 level.

b. Adjustment for multiple comparisons: Least Significant Difference (equivalent to no adjustments).

HC=0, MA use=1, MA dependence=2, MAP=3

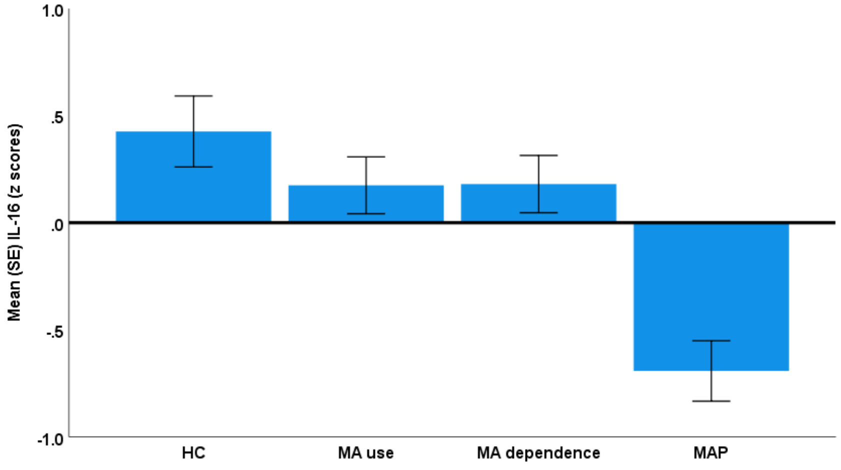

Figure S18. IL-16 levels in healthy controls (HC) and people with methamphetamine (MA) use, MA dependence, and MA-induced psychosis (MAP) (F=11.56, df=3/164, p<0.001).

Pairwise Comparisons

Dependent Variable: IL-16

(I) HC_Abuse_ Dependence_ Psychosis	(J) HC_Abuse_ Dependence_ Psychosis	Mean Difference (I-J)	Std. Error	Sig.[b]	95% Confidence Interval for Difference[b]	
					Lower Bound	Upper Bound
0	1	0.251	0.218	0.251	-0.179	0.682
	2	0.245	0.216	0.257	-0.181	0.672
	3	1.118*	0.215	0.000	0.694	1.542
1	0	-0.251	0.218	0.251	-0.682	0.179
	2	-0.006	0.188	0.975	-0.377	0.365
	3	.867*	0.199	0.000	0.473	1.260
2	0	-0.245	0.216	0.257	-0.672	0.181
	1	0.006	0.188	0.975	-0.365	0.377
	3	.873*	0.195	0.000	0.487	1.258
3	0	-1.118*	0.215	0.000	-1.542	-0.694
	1	-.867*	0.199	0.000	-1.260	-0.473
	2	-.873*	0.195	0.000	-1.258	-0.487

Based on estimated marginal means

*. The mean difference is significant at the 0.05 level.

b. Adjustment for multiple comparisons: Least Significant Difference (equivalent to no adjustments).

HC=0, MA use=1, MA dependence=2, MAP=3

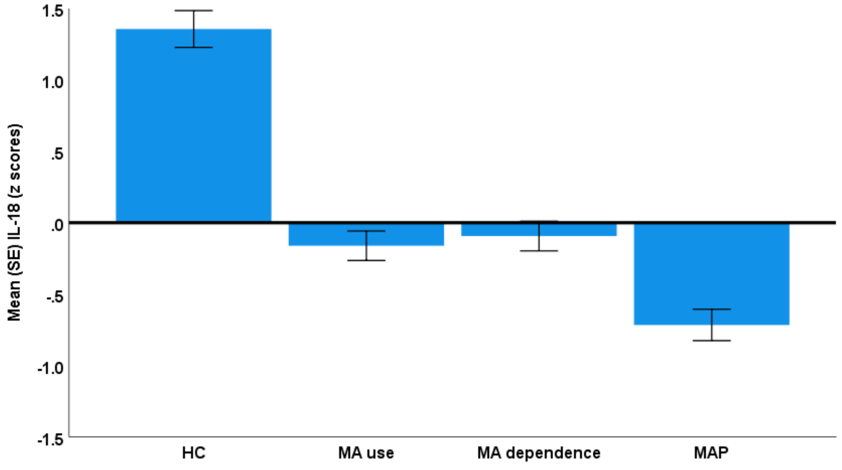

Figure S19. IL-18 levels in healthy controls (HC) and people with methamphetamine (MA) use, MA dependence, and MA-induced psychosis (MAP) (F=53.10, df=3/164, p<0.001).

Pairwise Comparisons

Dependent Variable: IL-18

(I) HC_Abuse_ Dependence_ Psychosis	(J) HC_Abuse_ Dependence_ Psychosis	Mean Difference (I-J)	Std. Error	Sig.[b]	95% Confidence Interval for Difference[b]	
					Lower Bound	Upper Bound
0	1	1.518*	0.170	0.000	1.183	1.853
	2	1.450*	0.168	0.000	1.118	1.782
	3	2.073*	0.167	0.000	1.743	2.403
1	0	-1.518*	0.170	0.000	-1.853	-1.183
	2	-0.068	0.146	0.643	-0.357	0.221
	3	.555*	0.155	0.000	0.248	0.862
2	0	-1.450*	0.168	0.000	-1.782	-1.118
	1	0.068	0.146	0.643	-0.221	0.357
	3	.623*	0.152	0.000	0.323	0.923
3	0	-2.073*	0.167	0.000	-2.403	-1.743
	1	-.555*	0.155	0.000	-0.862	-0.248
	2	-.623*	0.152	0.000	-0.923	-0.323

Based on estimated marginal means

*. The mean difference is significant at the 0.05 level.

b. Adjustment for multiple comparisons: Least Significant Difference (equivalent to no adjustments).

HC=0, MA use=1, MA dependence=2, MAP=3

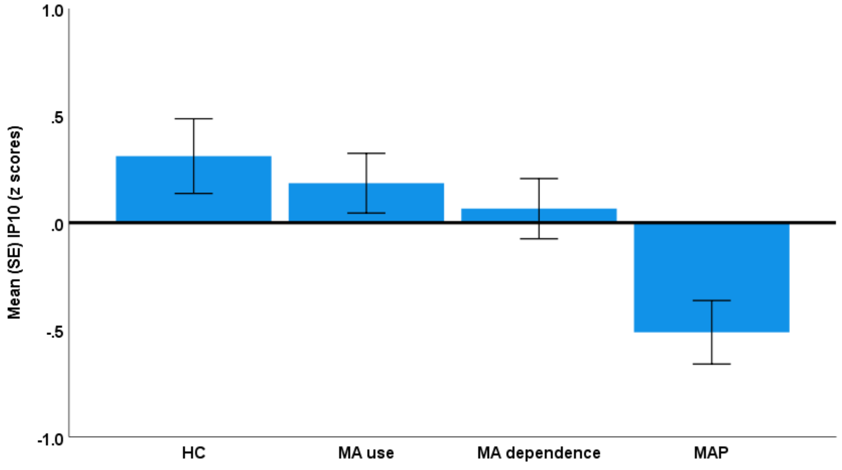

Figure S20. CXCL10 or IP10 levels in healthy controls (HC) and people with methamphetamine (MA) use, MA dependence, and MA-induced psychosis (MAP) (F=5.72, df=3/164, p<0.001).

Pairwise Comparisons

Dependent Variable: IP10

(I) HC_Abuse_Dependence_Psychosis	(J) HC_Abuse_Dependence_Psychosis	Mean Difference (I-J)	Std. Error	Sig.[b]	95% Confidence Interval for Difference[b]	
					Lower Bound	Upper Bound
0	1	0.127	0.229	0.582	-0.326	0.579
	2	0.246	0.227	0.281	-0.203	0.694
	3	.823*	0.226	0.000	0.377	1.269
1	0	-0.127	0.229	0.582	-0.579	0.326
	2	0.119	0.198	0.549	-0.272	0.509
	3	.696*	0.210	0.001	0.282	1.110
2	0	-0.246	0.227	0.281	-0.694	0.203
	1	-0.119	0.198	0.549	-0.509	0.272
	3	.577*	0.205	0.006	0.172	0.983
3	0	-.823*	0.226	0.000	-1.269	-0.377
	1	-.696*	0.210	0.001	-1.110	-0.282
	2	-.577*	0.205	0.006	-0.983	-0.172

Based on estimated marginal means

*. The mean difference is significant at the 0.05 level.

b. Adjustment for multiple comparisons: Least Significant Difference (equivalent to no adjustments).

HC=0, MA use=1, MA dependence=2, MAP=3

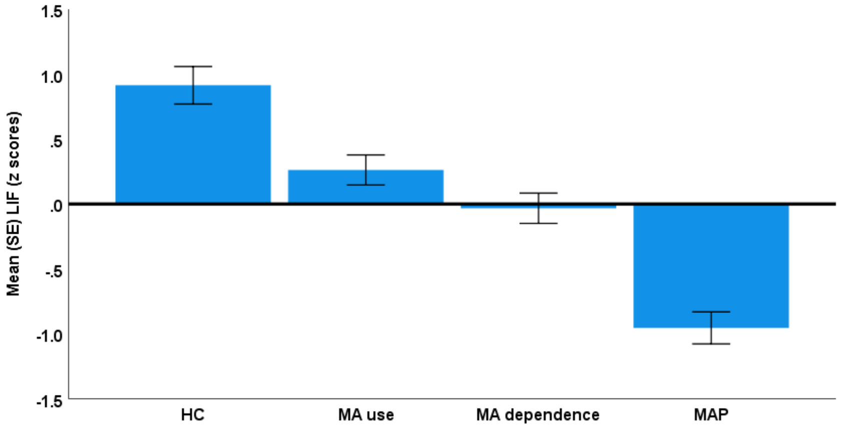

Figure S21. LIF levels in healthy controls (HC) and people with methamphetamine (MA) use, MA dependence, and MA-induced psychosis (MAP) (F=35.84, df=3/164, p<0.001).

Pairwise Comparisons

Dependent Variable: LIF

(I) HC_Abuse_ Dependence_ Psychosis	(J) HC_Abuse_ Dependence_ Psychosis	Mean Difference (I-J)	Std. Error	Sig.[b]	95% Confidence Interval for Difference[b]	
					Lower Bound	Upper Bound
0	1	.652*	0.190	0.001	0.276	1.028
	2	.947*	0.189	0.000	0.575	1.320
	3	1.869*	0.187	0.000	1.499	2.239
1	0	-.652*	0.190	0.001	-1.028	-0.276
	2	0.295	0.164	0.074	-0.029	0.619
	3	1.217*	0.174	0.000	0.873	1.560
2	0	-.947*	0.189	0.000	-1.320	-0.575
	1	-0.295	0.164	0.074	-0.619	0.029
	3	.921*	0.171	0.000	0.585	1.258
3	0	-1.869*	0.187	0.000	-2.239	-1.499
	1	-1.217*	0.174	0.000	-1.560	-0.873
	2	-.921*	0.171	0.000	-1.258	-0.585

Based on estimated marginal means

*. The mean difference is significant at the 0.05 level.

b. Adjustment for multiple comparisons: Least Significant Difference (equivalent to no adjustments).

HC=0, MA use=1, MA dependence=2, MAP=3

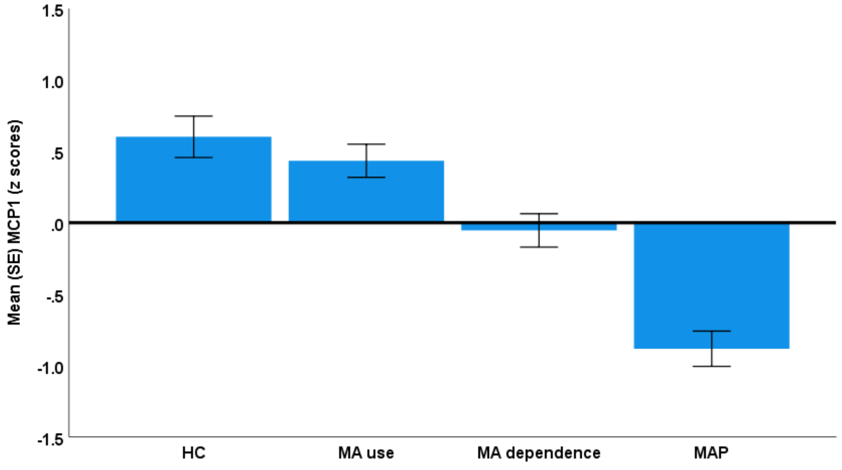

Figure S22. CCL2 (MCP1) levels in healthy controls (HC) and people with methamphetamine (MA) use, MA dependence, and MA-induced psychosis (MAP) (F=27.62, df=3/164, p<0.001).

Pairwise Comparisons

Dependent Variable: MCP1

(I) HC_Abuse_ Dependence_ Psychosis	(J) HC_Abuse_ Dependence_ Psychosis	Mean Difference (I-J)	Std. Error	Sig.[b]	95% Confidence Interval for Difference[b]	
					Lower Bound	Upper Bound
0	1	0.168	0.191	0.381	-0.209	0.545
	2	.656*	0.189	0.001	0.282	1.029
	3	1.485*	0.188	0.000	1.114	1.856
1	0	-0.168	0.191	0.381	-0.545	0.209
	2	.488*	0.165	0.003	0.163	0.813
	3	1.317*	0.175	0.000	0.972	1.662
2	0	-.656*	0.189	0.001	-1.029	-0.282
	1	-.488*	0.165	0.003	-0.813	-0.163
	3	.829*	0.171	0.000	0.491	1.167
3	0	-1.485*	0.188	0.000	-1.856	-1.114
	1	-1.317*	0.175	0.000	-1.662	-0.972
	2	-.829*	0.171	0.000	-1.167	-0.491

Based on estimated marginal means

*. The mean difference is significant at the 0.05 level.

b. Adjustment for multiple comparisons: Least Significant Difference (equivalent to no adjustments).

HC=0, MA use=1, MA dependence=2, MAP=3

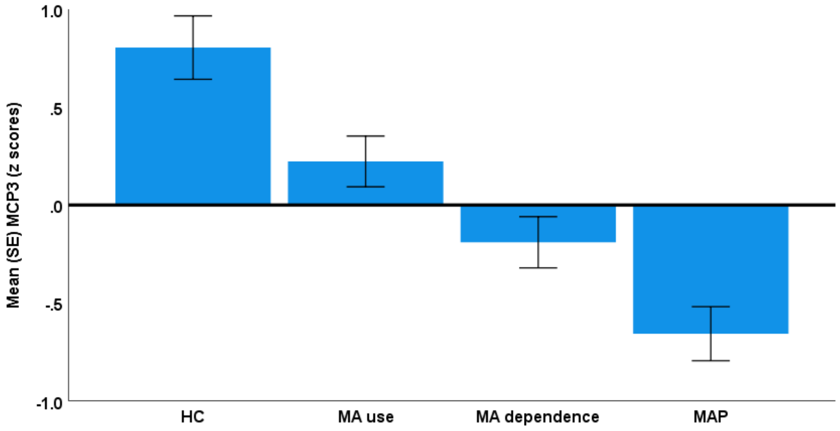

Figure S23. CCL7 (MCP3) levels in healthy controls (HC) and people with methamphetamine (MA) use, MA dependence, and MA-induced psychosis (MAP) (F=17.83, df=3/164, p<0.001).

Pairwise Comparisons

Dependent Variable: MCP3

(I) HC_Abuse_Dependence_Psychosis	(J) HC_Abuse_Dependence_Psychosis	Mean Difference (I-J)	Std. Error	Sig.[b]	95% Confidence Interval for Difference[b]	
					Lower Bound	Upper Bound
0	1	.582*	0.213	0.007	0.160	1.003
	2	.995*	0.211	0.000	0.578	1.412
	3	1.462*	0.210	0.000	1.047	1.876
1	0	-.582*	0.213	0.007	-1.003	-0.160
	2	.413*	0.184	0.026	0.050	0.776
	3	.880*	0.195	0.000	0.495	1.265
2	0	-.995*	0.211	0.000	-1.412	-0.578
	1	-.413*	0.184	0.026	-0.776	-0.050
	3	.467*	0.191	0.016	0.090	0.844
3	0	-1.462*	0.210	0.000	-1.876	-1.047
	1	-.880*	0.195	0.000	-1.265	-0.495
	2	-.467*	0.191	0.016	-0.844	-0.090

Based on estimated marginal means

*. The mean difference is significant at the 0.05 level.

b. Adjustment for multiple comparisons: Least Significant Difference (equivalent to no adjustments).

HC=0, MA use=1, MA dependence=2, MAP=3

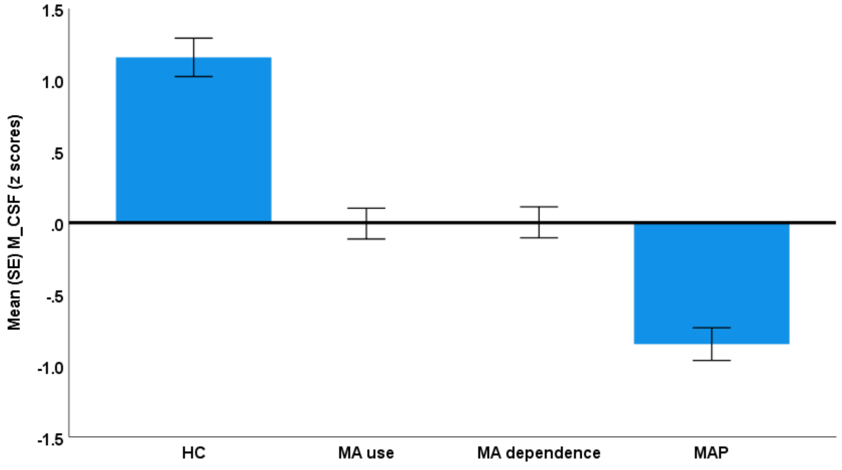

Figure S24. CSF levels in healthy controls (HC) and people with methamphetamine (MA) use, MA dependence, and MA-induced psychosis (MAP) (F=44.36, df=3/164, p<0.001).

Pairwise Comparisons

Dependent Variable: CSF

(I) HC_Abuse_ Dependence_ Psychosis	(J) HC_Abuse_ Dependence_ Psychosis	Mean Difference (I-J)	Std. Error	Sig.[b]	95% Confidence Interval for Difference[b]	
					Lower Bound	Upper Bound
0	1	1.164*	0.177	0.000	0.815	1.514
	2	1.156*	0.175	0.000	0.810	1.502
	3	2.009*	0.174	0.000	1.665	2.353
1	0	-1.164*	0.177	0.000	-1.514	-0.815
	2	-0.009	0.153	0.954	-0.310	0.293
	3	.844*	0.162	0.000	0.525	1.164
2	0	-1.156*	0.175	0.000	-1.502	-0.810
	1	0.009	0.153	0.954	-0.293	0.310
	3	.853*	0.159	0.000	0.540	1.166
3	0	-2.009*	0.174	0.000	-2.353	-1.665
	1	-.844*	0.162	0.000	-1.164	-0.525
	2	-.853*	0.159	0.000	-1.166	-0.540

Based on estimated marginal means

*. The mean difference is significant at the 0.05 level.

b. Adjustment for multiple comparisons: Least Significant Difference (equivalent to no adjustments).

HC=0, MA use=1, MA dependence=2, MAP=3

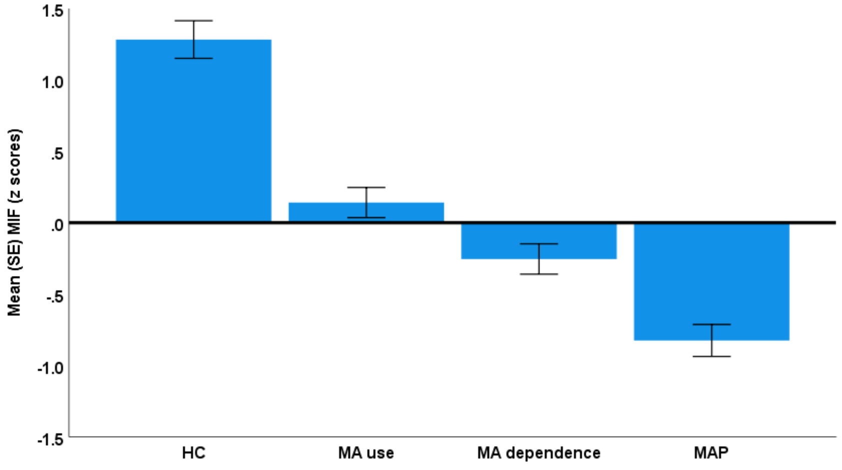

Figure S25. MIF levels in healthy controls (HC) and people with methamphetamine (MA) use, MA dependence, and MA-induced psychosis (MAP) (F=53.30, df=3/164, p<0.001).

Pairwise Comparisons

Dependent Variable: MIF

(I) HC_Abuse_ Dependence_ Psychosis	(J) HC_Abuse_ Dependence_ Psychosis	Mean Difference (I-J)	Std. Error	Sig.[b]	95% Confidence Interval for Difference[b]	
					Lower Bound	Upper Bound
0	1	1.141*	0.173	0.000	0.799	1.484
	2	1.537*	0.172	0.000	1.198	1.876
	3	2.107*	0.171	0.000	1.771	2.444
1	0	-1.141*	0.173	0.000	-1.484	-0.799
	2	.396*	0.149	0.009	0.100	0.691
	3	.966*	0.159	0.000	0.653	1.279
2	0	-1.537*	0.172	0.000	-1.876	-1.198
	1	-.396*	0.149	0.009	-0.691	-0.100
	3	.570*	0.155	0.000	0.264	0.877
3	0	-2.107*	0.171	0.000	-2.444	-1.771
	1	-.966*	0.159	0.000	-1.279	-0.653
	2	-.570*	0.155	0.000	-0.877	-0.264

Based on estimated marginal means

*. The mean difference is significant at the 0.05 level.

b. Adjustment for multiple comparisons: Least Significant Difference (equivalent to no adjustments).

HC=0, MA use=1, MA dependence=2, MAP=3

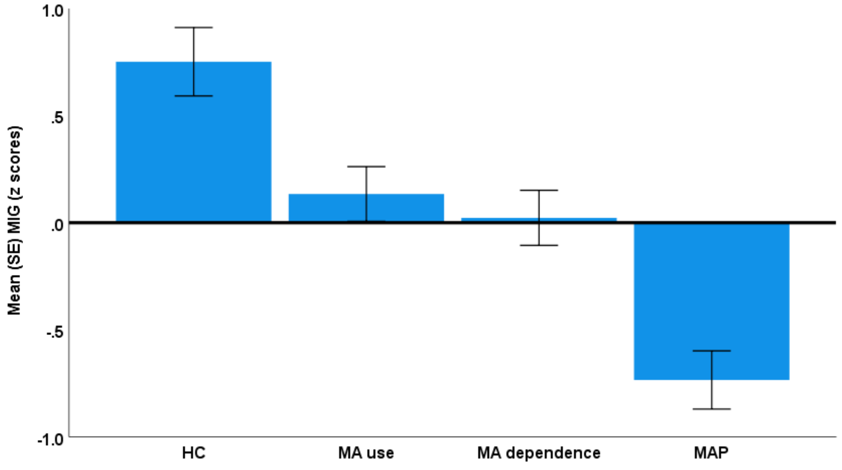

Figure S26. CXCL9 or MIG levels in healthy controls (HC) and people with methamphetamine (MA) use, MA dependence, and MA-induced psychosis (MAP) (F=17.96, df=3/164, p<0.001).

Pairwise Comparisons

Dependent Variable: MIG

(I) HC_Abuse_ Dependence_ Psychosis	(J) HC_Abuse_ Dependence_ Psychosis	Mean Difference (I-J)	Std. Error	Sig.[b]	95% Confidence Interval for Difference[b]	
					Lower Bound	Upper Bound
0	1	.618*	0.210	0.004	0.203	1.033
	2	.729*	0.208	0.001	0.318	1.140
	3	1.486*	0.207	0.000	1.078	1.894
1	0	-.618*	0.210	0.004	-1.033	-0.203
	2	0.111	0.181	0.540	-0.246	0.469
	3	.868*	0.192	0.000	0.489	1.247
2	0	-.729*	0.208	0.001	-1.140	-0.318
	1	-0.111	0.181	0.540	-0.469	0.246
	3	.757*	0.188	0.000	0.385	1.128
3	0	-1.486*	0.207	0.000	-1.894	-1.078
	1	-.868*	0.192	0.000	-1.247	-0.489
	2	-.757*	0.188	0.000	-1.128	-0.385

Based on estimated marginal means

*. The mean difference is significant at the 0.05 level.

b. Adjustment for multiple comparisons: Least Significant Difference (equivalent to no adjustments).

HC=0, MA use=1, MA dependence=2, MAP=3

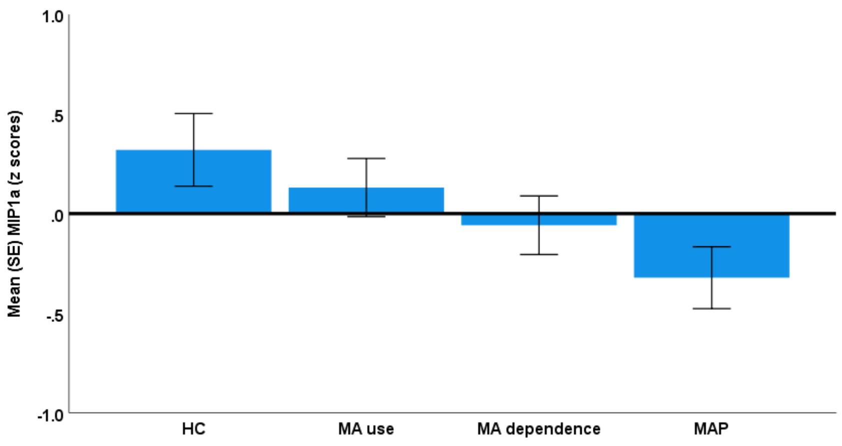

Figure S27. CCL3 or (MIP-1α) levels in healthy controls (HC) and people with methamphetamine (MA) use, MA dependence, and MA-induced psychosis (MAP) ($F=2.83$, $df=3/164$, $p=0.040$).

Pairwise Comparisons

Dependent Variable: MIP-1α

(I) HC_Abuse_ Dependence_ Psychosis	(J) HC_Abuse_ Dependence_ Psychosis	Mean Difference (I-J)	Std. Error	Sig.[b]	95% Confidence Interval for Difference[b]	
					Lower Bound	Upper Bound
0	1	0.189	0.240	0.431	-0.285	0.663
	2	0.378	0.238	0.113	-0.091	0.848
	3	.642*	0.236	0.007	0.175	1.108
1	0	-0.189	0.240	0.431	-0.663	0.285
	2	0.189	0.207	0.362	-0.220	0.598
	3	.452*	0.219	0.041	0.019	0.886
2	0	-0.378	0.238	0.113	-0.848	0.091
	1	-0.189	0.207	0.362	-0.598	0.220
	3	0.263	0.215	0.222	-0.161	0.688
3	0	-.642*	0.236	0.007	-1.108	-0.175
	1	-.452*	0.219	0.041	-0.886	-0.019
	2	-0.263	0.215	0.222	-0.688	0.161

Based on estimated marginal means

*. The mean difference is significant at the 0.05 level.

b. Adjustment for multiple comparisons: Least Significant Difference (equivalent to no adjustments).

HC=0, MA use=1, MA dependence=2, MAP=3

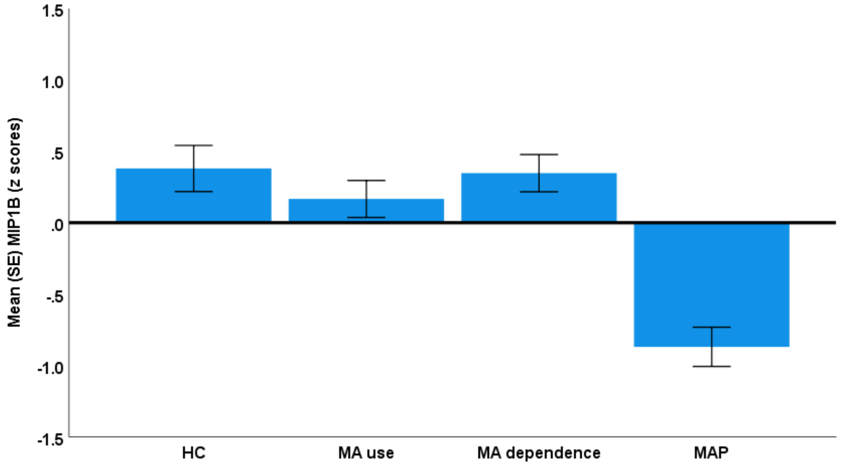

Figure S28. CCL4 or MIP-1β levels in healthy controls (HC) and people with methamphetamine (MA) use, MA dependence, and MA-induced psychosis (MAP) (F=18.20, df=3/164, p<0.001).

Pairwise Comparisons

Dependent Variable: MIP-1β

(I) HC_Abuse_ Dependence_ Psychosis	(J) HC_Abuse_ Dependence_ Psychosis	Mean Difference (I-J)	Std. Error	Sig.[b]	95% Confidence Interval for Difference[b]	
					Lower Bound	Upper Bound
0	1	0.213	0.213	0.317	-0.206	0.633
	2	0.033	0.211	0.877	-0.383	0.448
	3	1.249*	0.209	0.000	0.836	1.662
1	0	-0.213	0.213	0.317	-0.633	0.206
	2	-0.181	0.183	0.326	-0.543	0.181
	3	1.036*	0.194	0.000	0.652	1.419
2	0	-0.033	0.211	0.877	-0.448	0.383
	1	0.181	0.183	0.326	-0.181	0.543
	3	1.216*	0.190	0.000	0.840	1.592
3	0	-1.249*	0.209	0.000	-1.662	-0.836
	1	-1.036*	0.194	0.000	-1.419	-0.652
	2	-1.216*	0.190	0.000	-1.592	-0.840

Based on estimated marginal means

*. The mean difference is significant at the 0.05 level.

b. Adjustment for multiple comparisons: Least Significant Difference (equivalent to no adjustments).

HC=0, MA use=1, MA dependence=2, MAP=3

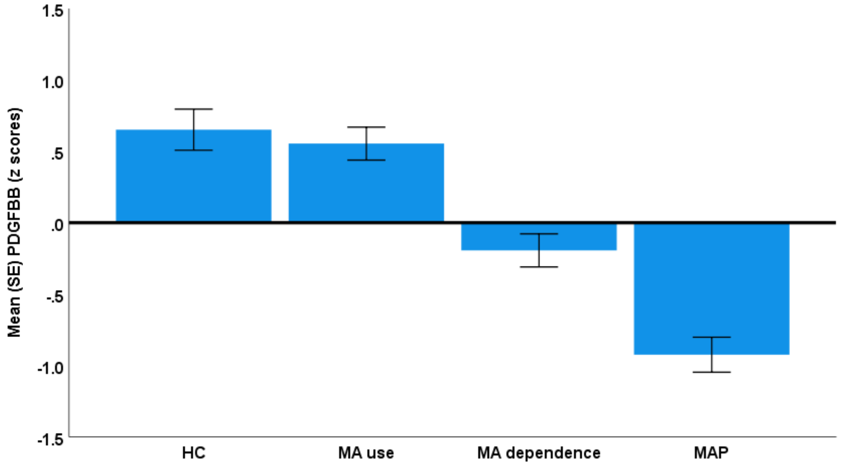

Figure S29. PDGF-BB levels in healthy controls (HC) and people with methamphetamine (MA) use, MA dependence, and MA-induced psychosis (MAP) (F=34.46, df=3/164, p<0.001).

Pairwise Comparisons

Dependent Variable: PDGFBB

(I) HC_Abuse_Dependence_Psychosis	(J) HC_Abuse_Dependence_Psychosis	Mean Difference (I-J)	Std. Error	Sig.[b]	95% Confidence Interval for Difference[b]	
					Lower Bound	Upper Bound
0	1	0.097	0.189	0.608	-0.277	0.472
	2	.845*	0.188	0.000	0.475	1.216
	3	1.576*	0.186	0.000	1.208	1.944
1	0	-0.097	0.189	0.608	-0.472	0.277
	2	.748*	0.163	0.000	0.425	1.070
	3	1.479*	0.173	0.000	1.137	1.821
2	0	-.845*	0.188	0.000	-1.216	-0.475
	1	-.748*	0.163	0.000	-1.070	-0.425
	3	.731*	0.170	0.000	0.396	1.066
3	0	-1.576*	0.186	0.000	-1.944	-1.208
	1	-1.479*	0.173	0.000	-1.821	-1.137
	2	-.731*	0.170	0.000	-1.066	-0.396

Based on estimated marginal means

*. The mean difference is significant at the 0.05 level.

b. Adjustment for multiple comparisons: Least Significant Difference (equivalent to no adjustments).

HC=0, MA use=1, MA dependence=2, MAP=3

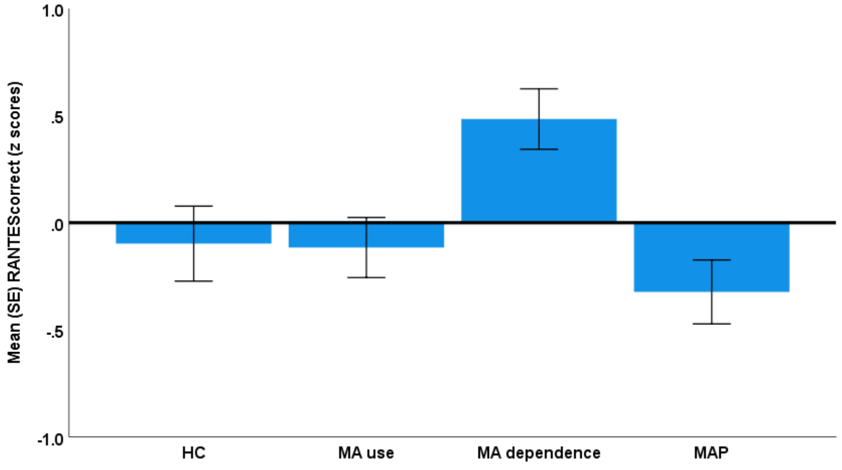

Figure S30. CCL5 or RANTES levels in healthy controls (HC) and people with methamphetamine (MA) use, MA dependence, and MA-induced psychosis (MAP) (F=5.81, df=3/164, p<0.001).

Pairwise Comparisons

Dependent Variable: RANTES

(I) HC_Abuse_ Dependence_ Psychosis	(J) HC_Abuse_ Dependence_ Psychosis	Mean Difference (I-J)	Std. Error	Sig.[b]	95% Confidence Interval for Difference[b]	
					Lower Bound	Upper Bound
0	1	0.018	0.231	0.937	-0.437	0.473
	2	-.582*	0.228	0.012	-1.033	-0.131
	3	0.226	0.227	0.321	-0.222	0.674
1	0	-0.018	0.231	0.937	-0.473	0.437
	2	-.600*	0.199	0.003	-0.993	-0.207
	3	0.207	0.211	0.327	-0.209	0.624
2	0	.582*	0.228	0.012	0.131	1.033
	1	.600*	0.199	0.003	0.207	0.993
	3	.807*	0.207	0.000	0.400	1.215
3	0	-0.226	0.227	0.321	-0.674	0.222
	1	-0.207	0.211	0.327	-0.624	0.209
	2	-.807*	0.207	0.000	-1.215	-0.400

Based on estimated marginal means

*. The mean difference is significant at the 0.05 level.

b. Adjustment for multiple comparisons: Least Significant Difference (equivalent to no adjustments).

HC=0, MA use=1, MA dependence=2, MAP=3

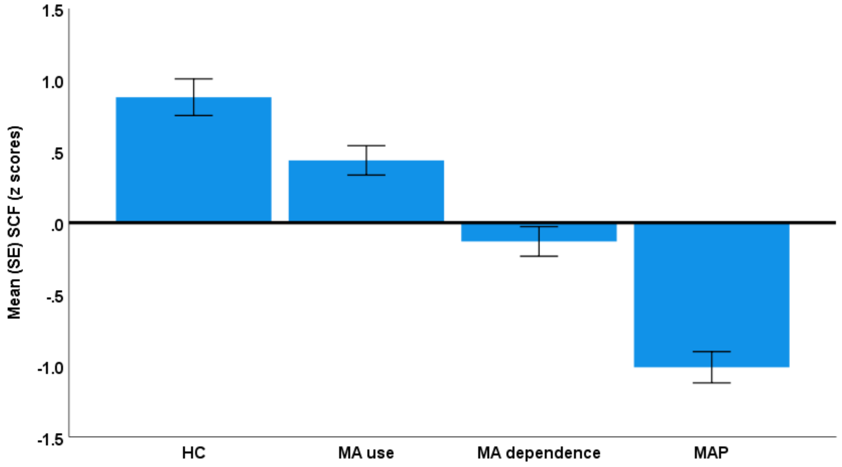

Figure S31. SCF levels in healthy controls (HC) and people with methamphetamine (MA) use, MA dependence, and MA-induced psychosis (MAP) ($F=51.39$, df=3/164, $p<0.001$).

Pairwise Comparisons

Dependent Variable: SCF

(I) HC_Abuse_ Dependence_ Psychosis	(J) HC_Abuse_ Dependence_ Psychosis	Mean Difference (I-J)	Std. Error	Sig.[b]	95% Confidence Interval for Difference[b]	
					Lower Bound	Upper Bound
0	1	.443*	0.169	0.010	0.109	0.776
	2	1.010*	0.167	0.000	0.679	1.341
	3	1.892*	0.166	0.000	1.563	2.220
1	0	-.443*	0.169	0.010	-0.776	-0.109
	2	.567*	0.146	0.000	0.280	0.855
	3	1.449*	0.155	0.000	1.144	1.754
2	0	-1.010*	0.167	0.000	-1.341	-0.679
	1	-.567*	0.146	0.000	-0.855	-0.280
	3	.882*	0.151	0.000	0.582	1.181
3	0	-1.892*	0.166	0.000	-2.220	-1.563
	1	-1.449*	0.155	0.000	-1.754	-1.144
	2	-.882*	0.151	0.000	-1.181	-0.582

Based on estimated marginal means

*. The mean difference is significant at the 0.05 level.

b. Adjustment for multiple comparisons: Least Significant Difference (equivalent to no adjustments).

HC=0, MA use=1, MA dependence=2, MAP=3

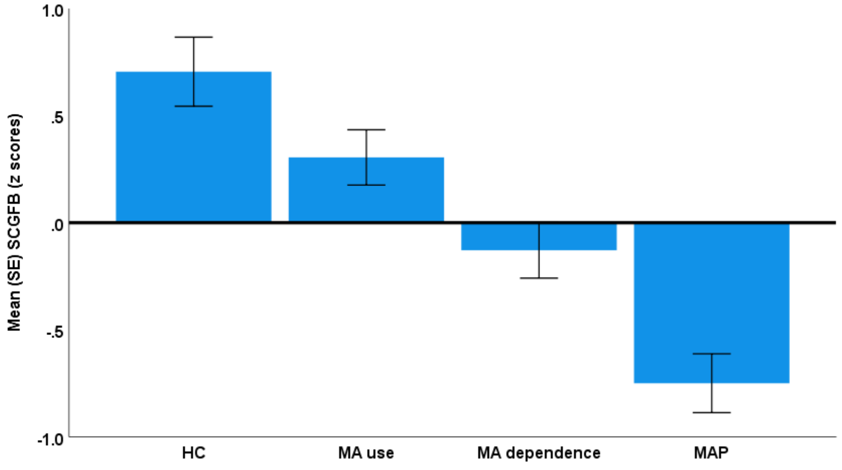

Figure S32. SCGF-β levels in healthy controls (HC) and people with methamphetamine (MA) use, MA dependence, and MA-induced psychosis (MAP) ($F=18.79$, $df=3/164$, $p<0.001$).

Pairwise Comparisons

Dependent Variable: SCGF-β

(I) HC_Abuse_ Dependence_ Psychosis	(J) HC_Abuse_ Dependence_ Psychosis	Mean Difference (I-J)	Std. Error	Sig.[b]	95% Confidence Interval for Difference[b]	
					Lower Bound	Upper Bound
0	1	0.400	0.212	0.061	-0.019	0.819
	2	.834*	0.210	0.000	0.419	1.249
	3	1.454*	0.209	0.000	1.042	1.867
1	0	-0.400	0.212	0.061	-0.819	0.019
	2	.434*	0.183	0.019	0.072	0.795
	3	1.054*	0.194	0.000	0.671	1.437
2	0	-.834*	0.210	0.000	-1.249	-0.419
	1	-.434*	0.183	0.019	-0.795	-0.072
	3	.621*	0.190	0.001	0.245	0.996
3	0	-1.454*	0.209	0.000	-1.867	-1.042
	1	-1.054*	0.194	0.000	-1.437	-0.671
	2	-.621*	0.190	0.001	-0.996	-0.245

Based on estimated marginal means

*. The mean difference is significant at the 0.05 level.

b. Adjustment for multiple comparisons: Least Significant Difference (equivalent to no adjustments).

HC=0, MA use=1, MA dependence=2, MAP=3

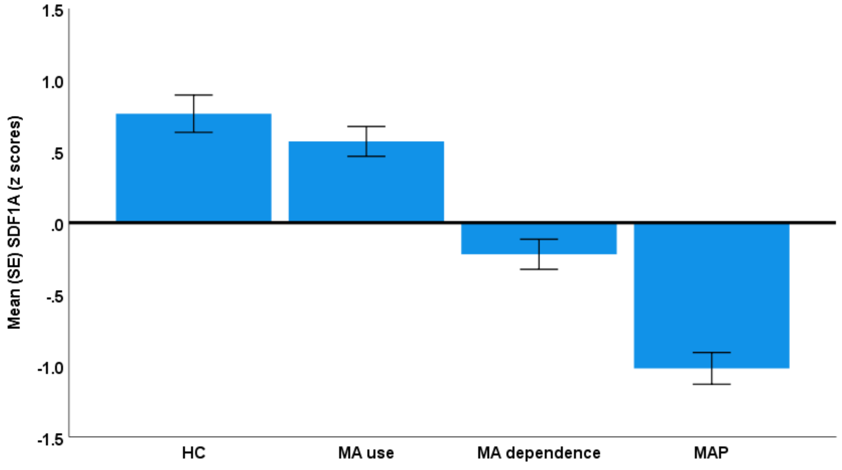

Figure S33. CXCL12 or SDF-1α levels in healthy controls (HC) and people with methamphetamine (MA) use, MA dependence, and MA-induced psychosis (MAP) (F=50.94, df=3/164, p<0.001).

Pairwise Comparisons

Dependent Variable: SDF-1α

(I) HC_Abuse_ Dependence_ Psychosis	(J) HC_Abuse_ Dependence_ Psychosis	Mean Difference (I-J)	Std. Error	Sig.[b]	95% Confidence Interval for Difference[b]	
					Lower Bound	Upper Bound
0	1	0.194	0.172	0.260	-0.145	0.534
	2	.985*	0.170	0.000	0.648	1.321
	3	1.784*	0.169	0.000	1.450	2.118
1	0	-0.194	0.172	0.260	-0.534	0.145
	2	.790*	0.148	0.000	0.497	1.083
	3	1.590*	0.157	0.000	1.279	1.900
2	0	-.985*	0.170	0.000	-1.321	-0.648
	1	-.790*	0.148	0.000	-1.083	-0.497
	3	.799*	0.154	0.000	0.495	1.103
3	0	-1.784*	0.169	0.000	-2.118	-1.450
	1	-1.590*	0.157	0.000	-1.900	-1.279
	2	-.799*	0.154	0.000	-1.103	-0.495

Based on estimated marginal means

*. The mean difference is significant at the 0.05 level.

b. Adjustment for multiple comparisons: Least Significant Difference (equivalent to no adjustments).

HC=0, MA use=1, MA dependence=2, MAP=3

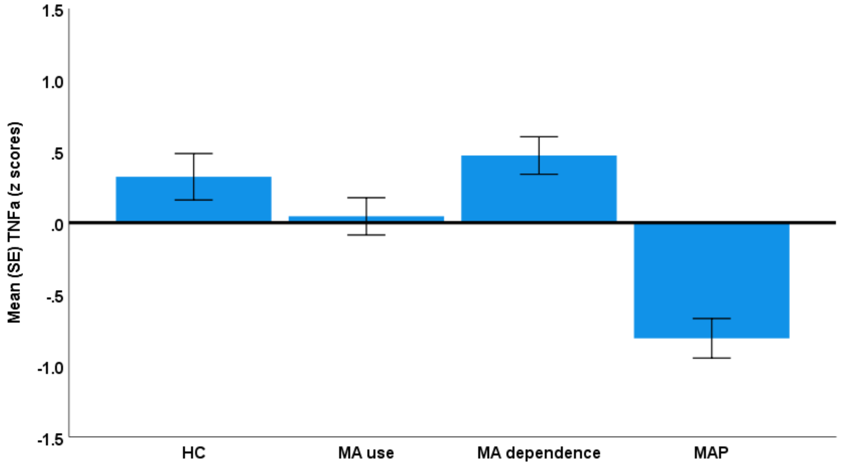

Figure S34. TNF-α levels in healthy controls (HC) and people with methamphetamine (MA) use, MA dependence, and MA-induced psychosis (MAP) (F=17.20, df=3/164, p<0.001).

Pairwise Comparisons

Dependent Variable: TNF-α

(I) HC_Abuse_ Dependence_ Psychosis	(J) HC_Abuse_ Dependence_ Psychosis	Mean Difference (I-J)	Std. Error	Sig.[b]	95% Confidence Interval for Difference[b]	
					Lower Bound	Upper Bound
0	1	0.277	0.214	0.197	-0.146	0.700
	2	-0.149	0.212	0.484	-0.568	0.270
	3	1.131*	0.211	0.000	0.715	1.548
1	0	-0.277	0.214	0.197	-0.700	0.146
	2	-.426*	0.185	0.022	-0.791	-0.061
	3	.854*	0.196	0.000	0.467	1.241
2	0	0.149	0.212	0.484	-0.270	0.568
	1	.426*	0.185	0.022	0.061	0.791
	3	1.280*	0.192	0.000	0.901	1.659
3	0	-1.131*	0.211	0.000	-1.548	-0.715
	1	-.854*	0.196	0.000	-1.241	-0.467
	2	-1.280*	0.192	0.000	-1.659	-0.901

Based on estimated marginal means

*. The mean difference is significant at the 0.05 level.

b. Adjustment for multiple comparisons: Least Significant Difference (equivalent to no adjustments).

HC=0, MA use=1, MA dependence=2, MAP=3

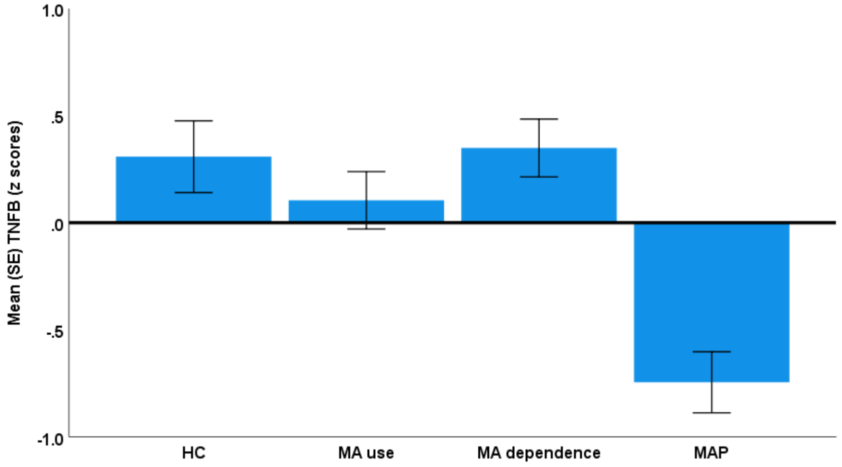

Figure S35. TNF-β levels in healthy controls (HC) and people with methamphetamine (MA) use, MA dependence, and MA-induced psychosis (MAP) ($F=12.78$, $df=3/164$, $p<0.001$).

Pairwise Comparisons

Dependent Variable: TNF-β

(I) HC_Abuse_ Dependence_ Psychosis	(J) HC_Abuse_ Dependence_ Psychosis	Mean Difference (I-J)	Std. Error	Sig.[b]	95% Confidence Interval for Difference[b]	
					Lower Bound	Upper Bound
0	1	0.204	0.220	0.357	-0.231	0.639
	2	-0.041	0.218	0.853	-0.471	0.390
	3	1.053*	0.217	0.000	0.626	1.481
1	0	-0.204	0.220	0.357	-0.639	0.231
	2	-0.244	0.190	0.200	-0.619	0.131
	3	.850*	0.201	0.000	0.452	1.248
2	0	0.041	0.218	0.853	-0.390	0.471
	1	0.244	0.190	0.200	-0.131	0.619
	3	1.094*	0.197	0.000	0.704	1.484
3	0	-1.053*	0.217	0.000	-1.481	-0.626
	1	-.850*	0.201	0.000	-1.248	-0.452
	2	-1.094*	0.197	0.000	-1.484	-0.704

Based on estimated marginal means

*. The mean difference is significant at the 0.05 level.

b. Adjustment for multiple comparisons: Least Significant Difference (equivalent to no adjustments).

HC=0, MA use=1, MA dependence=2, MAP=3

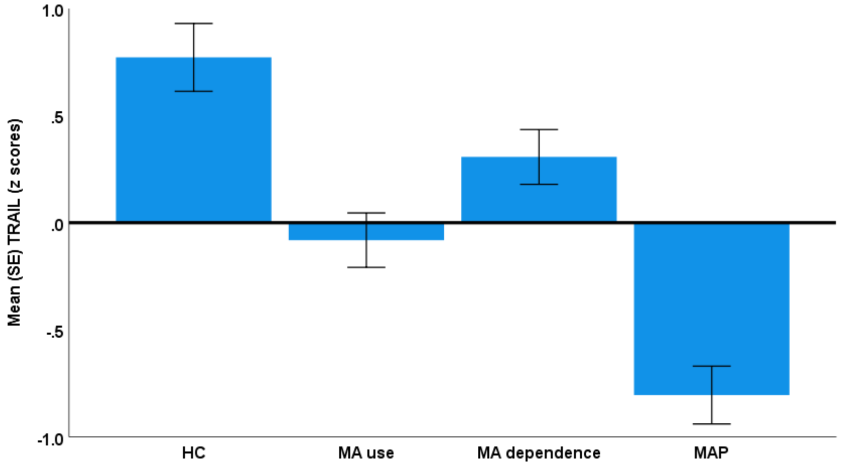

Figure S36. TRAIL levels in healthy controls (HC) and people with methamphetamine (MA) use, MA dependence, and MA-induced psychosis (MAP) (F=22.53, df=3/164, p<0.001).

Pairwise Comparisons

Dependent Variable: TRAIL

(I) HC_Abuse_ Dependence_ Psychosis	(J) HC_Abuse_ Dependence_ Psychosis	Mean Difference (I-J)	Std. Error	Sig.[b]	95% Confidence Interval for Difference[b]	
					Lower Bound	Upper Bound
0	1	.854*	0.209	0.000	0.441	1.266
	2	.465*	0.207	0.026	0.057	0.873
	3	1.577*	0.205	0.000	1.171	1.982
1	0	-.854*	0.209	0.000	-1.266	-0.441
	2	-.389*	0.180	0.032	-0.744	-0.033
	3	.723*	0.191	0.000	0.346	1.100
2	0	-.465*	0.207	0.026	-0.873	-0.057
	1	.389*	0.180	0.032	0.033	0.744
	3	1.112*	0.187	0.000	0.743	1.481
3	0	-1.577*	0.205	0.000	-1.982	-1.171
	1	-.723*	0.191	0.000	-1.100	-0.346
	2	-1.112*	0.187	0.000	-1.481	-0.743

Based on estimated marginal means

*. The mean difference is significant at the 0.05 level.

b. Adjustment for multiple comparisons: Least Significant Difference (equivalent to no adjustments).

HC=0, MA use=1, MA dependence=2, MAP=3
